# Functional expression of foreign magnetosome genes in the alphaproteobacterium *Magnetospirillum gryphiswaldense*

**DOI:** 10.1128/mbio.03282-22

**Published:** 2023-06-15

**Authors:** Ram Prasad Awal, Christopher T. Lefevre, Dirk Schüler

**Affiliations:** 1 Department of Microbiology, University of Bayreuth, Bayreuth, Germany; 2 Aix-Marseille Université, CEA, CNRS, Institute of Biosciences and Biotechnologies of Aix-Marseille, Saint-Paul-lez-Durance, France; Korea Advanced Institute of Science and Technology, Yuseong-gu, Daejeon, South Korea

**Keywords:** magnetotactic bacteria, orthologues, heterologous expression, TAR cloning, MGC

## Abstract

Magnetosomes of magnetotactic bacteria (MTB) consist of structurally perfect, nano-sized magnetic crystals enclosed within vesicles of a proteo-lipid membrane. In species of *Magnetospirillum,* biosynthesis of their cubo-octahedral-shaped magnetosomes was recently demonstrated to be a complex process, governed by about 30 specific genes that are comprised within compact magnetosome gene clusters (MGCs). Similar, yet distinct gene clusters were also identified in diverse MTB that biomineralize magnetosome crystals with different, genetically encoded morphologies. However, since most representatives of these groups are inaccessible by genetic and biochemical approaches, their analysis will require the functional expression of magnetosome genes in foreign hosts. Here, we studied whether conserved essential magnetosome genes from closely and remotely related MTB can be functionally expressed by rescue of their respective mutants in the tractable model *Magnetospirillum gryphiswaldense* of the *Alphaproteobacteria*. Upon chromosomal integration, single orthologues from other magnetotactic *Alphaproteobacteria* restored magnetosome biosynthesis to different degrees, while orthologues from distantly related *Magnetococcia* and *Deltaproteobacteria* were found to be expressed but failed to re-induce magnetosome biosynthesis, possibly due to poor interaction with their cognate partners within multiprotein magnetosome organelle of the host. Indeed, co-expression of the known interactors MamB and MamM from the alphaproteobacterium *Magnetovibrio blakemorei* increased functional complementation. Furthermore, a compact and portable version of the entire MGCs of *M. magneticum* was assembled by transformation-associated recombination cloning, and it restored the ability to biomineralize magnetite both in deletion mutants of the native donor and *M. gryphiswaldense*, while co-expression of gene clusters from both *M. gryphiswaldense* and *M. magneticum* resulted in overproduction of magnetosomes.

**IMPORTANCE:**

We provide proof of principle that *Magnetospirillum gryphiswaldense* is a suitable surrogate host for the functional expression of foreign magnetosome genes and extended the transformation-associated recombination cloning platform for the assembly of entire large magnetosome gene cluster, which could then be transplanted to different magnetotactic bacteria. The reconstruction, transfer, and analysis of gene sets or entire magnetosome clusters will be also promising for engineering the biomineralization of magnetite crystals with different morphologies that would be valuable for biotechnical applications.

## INTRODUCTION

Magnetotactic bacteria (MTB) are a diverse group of prokaryotes able to navigate within the Earth’s magnetic field by specific organelles. These so-called magnetosomes are membrane-enclosed crystals of a magnetic iron mineral, which serve as intracellular sensors that are thought to direct the aerotactic swimming motility along vertical redox gradients in the aquatic sediments, where MTB occur abundantly and ubiquitously (1 - 3). In the well-studied alphaproteobacterium *Magnetospirillum gryphiswaldense* (MSR-1) and closely related MTB, biosynthesis of magnetosomes has recently been demonstrated to be a rather intricate step-wise process, which is initiated by the formation of magnetosome vesicles by invagination from the cytoplasmic membrane (CM). This is followed by the magnetosomal uptake of large amounts of iron that becomes mineralized as monocrystalline particles of magnetite (Fe_3_O_4_) having a regular cubo-octahedral shape and uniform size of about 45 nm in their mature state (4 - 8). Nascent magnetosome crystals become concatenated into linear magnetosome chains that are assembled, positioned, and partitioned by a dedicated multipartite cytoskeleton (magnetoskeleton) (2, 9, 10). In MSR-1 and the other few magnetospirilla that have been experimentally analyzed, all these steps were found to be orchestrated and tightly controlled by more than 30 magnetosome-associated proteins designated as Mam (magnetosome membrane), Mms (magnetosome membrane specific), and Feo (ferrous iron transport) system (11 - 14). These are encoded by genes of several operons designated *mamAB*op, *mamGFDC*op, *mms6*op, *mamXY*op, and *FeoAB*, also referred to as magnetosome gene clusters (MGCs) (15) located within a genomic “magnetosome island” (MAI) (16 - 18).

In contrast to the comparably simple isotropic cubo-octahedral crystals of magnetite (Fe_3_O_4_) produced by *Magnetospirillum* spp., many other MTB species display a spectacular diversity with respect to composition, shapes, sizes, number, and intracellular organization of magnetosomes (19 - 21). For example, many magnetotactic alpha-, beta-, eta-, and gamma-proteobacteria biomineralize magnetite crystals with elongated prismatic morphologies, while magnetotactic *Deltaproteobacteria*, *Nitrospirae*, and *Omnitrophica* form bullet-shaped crystals of magnetite, greigite (Fe_3_S_4_), or both (15, 20, 22 - 28). Genomics and metagenomics revealed that MGCs akin to the MAI of magnetospirilla are present in apparently all of the numerous species of MTB (15, 24, 25, 27, 29). However, these MGCs are diverse with respect to sequence identity as well as gene content, and a set of only few core genes (*mamABEKMLOPQI*) is common to most magnetite-producing MTB (15, 23), while even fewer (*mamABIKMQ*) might be conserved in all MTB (26, 27). The astonishing diversity of magnetosome morphologies as well as their diverse MGCs indicates that magnetosome biogenesis pathways are somewhat divergent, and it has been speculated that the observed genetic diversity likely accounts for the biomineralization of morphologically distinct magnetosome crystals by as yet unknown mechanisms (27, 30 - 32). As different morphologies are also expected to be associated with different magnetic properties, understanding the diverse pathways of magnetosomes formation is also of biotechnological interest for the use of magnetosomes as biogenic magnetic nanoparticles (33 - 36). However, since MTB forming magnetosome crystals with shapes other than cubo-octahedral are not, or only poorly amenable (37) to genetic analysis, or cannot be cultured in the lab at all (22, 38), it has remained entirely elusive how diverse MTB control the biomineralization of elongated or bullet-shaped magnetosome at the genetic, biochemical, and structural level. Therefore, the experimental elucidation of genetic functions and diverse mechanisms of magnetosome biosynthesis will require alternative approaches such as the heterologous expression of genes in more tractable surrogate hosts, which has proven as a powerful strategy for the expression of genes and clusters for other bacterial products (39).

**Fig 1:**
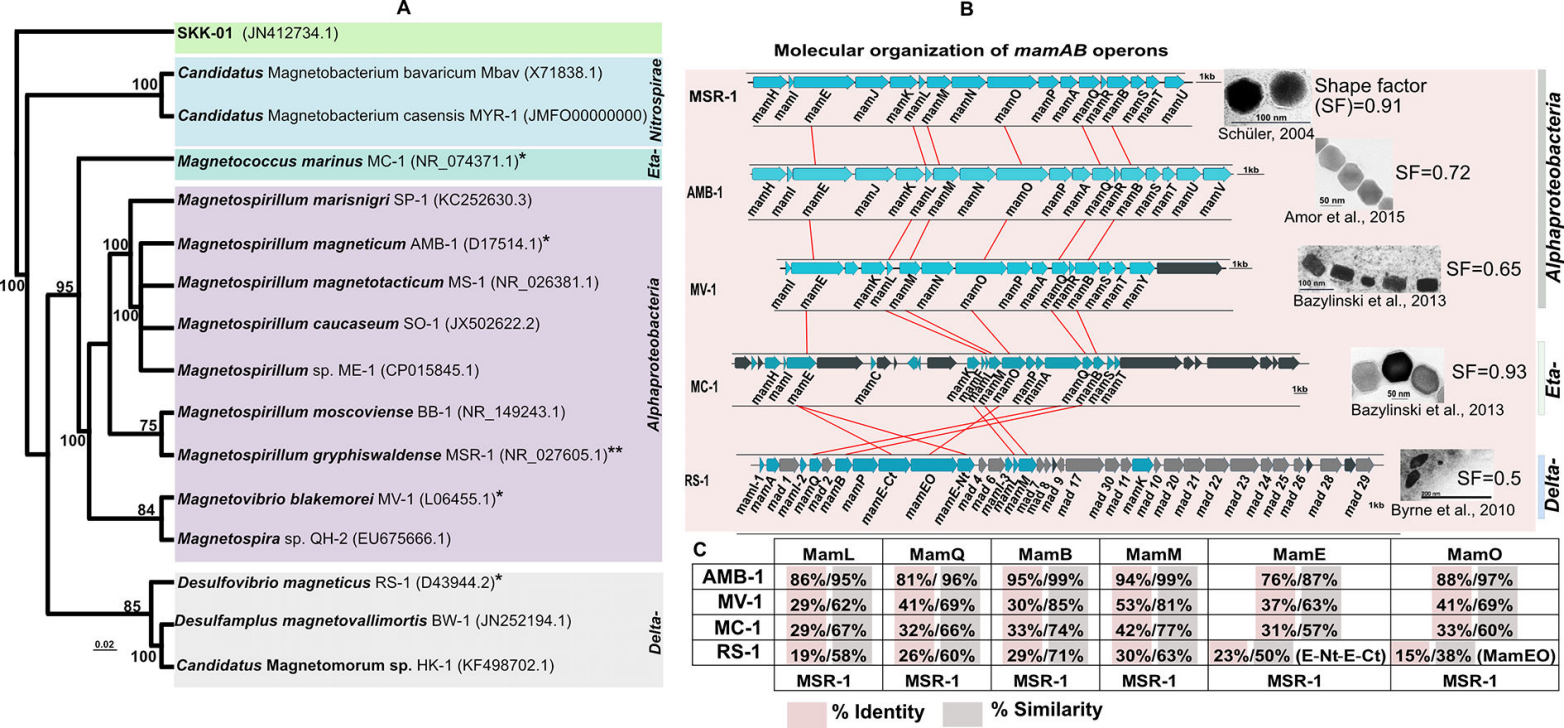
(**A**) Neighbor-joining tree of magnetotactic bacteria based on 16S rRNA gene sequences showing the position of the host (indicated **) and donor strain (indicated *) used in this study. GenBank accession numbers are shown in parentheses. *Omnitrophica* SKK-01 was used as an outgroup to root the tree. Bar, 0.02 substitutions per nucleotide position. (**B**) Molecular organization of genes in the *mamAB* operons of MSR-1, AMB-1, MV-1, MC-1, and RS-1. Homologous genes selected for this study are connected by red lines. Blue arrows indicate genes known to be involved in magnetosome biosynthesis, black arrows are genes of unknown function within *mamAB* operons, and gray arrows indicate known *mad* genes in the deltaproteobacterium RS-1. The SF of magnetosomes is a ratio of width to length. (**C**) % Identity/% similarity of Mam proteins from donor strains to that of MSR-1.

Because of its tractability (40 - 42) and relatively straightforward cultivation (43), MSR-1 has emerged as a model in many studies on the biosynthesis of magnetosomes (44), and it has been further optimized for enhanced robustness, growth, and the stable expression of native and foreign genes by engineering of “chassis” strains (45, 46). However, with few exceptions (e.g., *mamK* and *mamB* orthologues from closely related species [47, 48]), it has not been tested experimentally whether magnetosome genes from MTB with diverse magnetosome morphologies can be functionally expressed in MSR-1. To address this question, we studied whether conserved magnetosome genes and entire MGCs from various foreign MTB can substitute the functions of MSR-1 orthologues by complementation of isogenic deletions. As donors, we choose several cultured MTB, which cover a range of phylogenetic divergence from three bacterial classes: the *Alphaproteobacteria* including the closely related *M. magneticum* AMB-1 and the more remotely related *Magnetovibrio blakemorei* MV-1, the *Deltaproteobacteria* with *Desulfovibrio magneticus* RS-1, and the candidate class *Magnetococcia* (49) with *Magnetococcus marinus* MC-1 (Fig. 1A). We show that while orthologues from remotely related MTB are poorly or not functional, single orthologues and entire MGCs from more closely related MTB can fully restore magnetite biomineralization in MSR-1.

## RESULTS

### Expression of single foreign magnetosome gene in MSR-1

We first studied whether orthologues of single magnetosome (*mam*) genes from different MTB can rescue respective mutants in MSR-1. The donor AMB-1 produces roughly cubo-octahedral but slightly more elongated magnetite crystals (50) than MSR-1, which unlike in MSR-1 are arranged in fragmented chains separated by gaps due to the presence of empty vesicles that do not contain magnetite crystals (51, 52). The vibroid marine MV-1 produces about 10 pseudo-hexagonal elongated prismatic magnetite crystals per cell (53, 54). RS-1 is a freshwater sulfate-reducing bacterium (55) that produces 12–15 irregular bullet-shaped magnetite crystals aligned in a chain (56). Magnetosomes from the marine MC-1 are aligned in a single chain of 10–14 elongated pseudo-hexagonal prismatic magnetite crystals (57).

**Fig 2:**
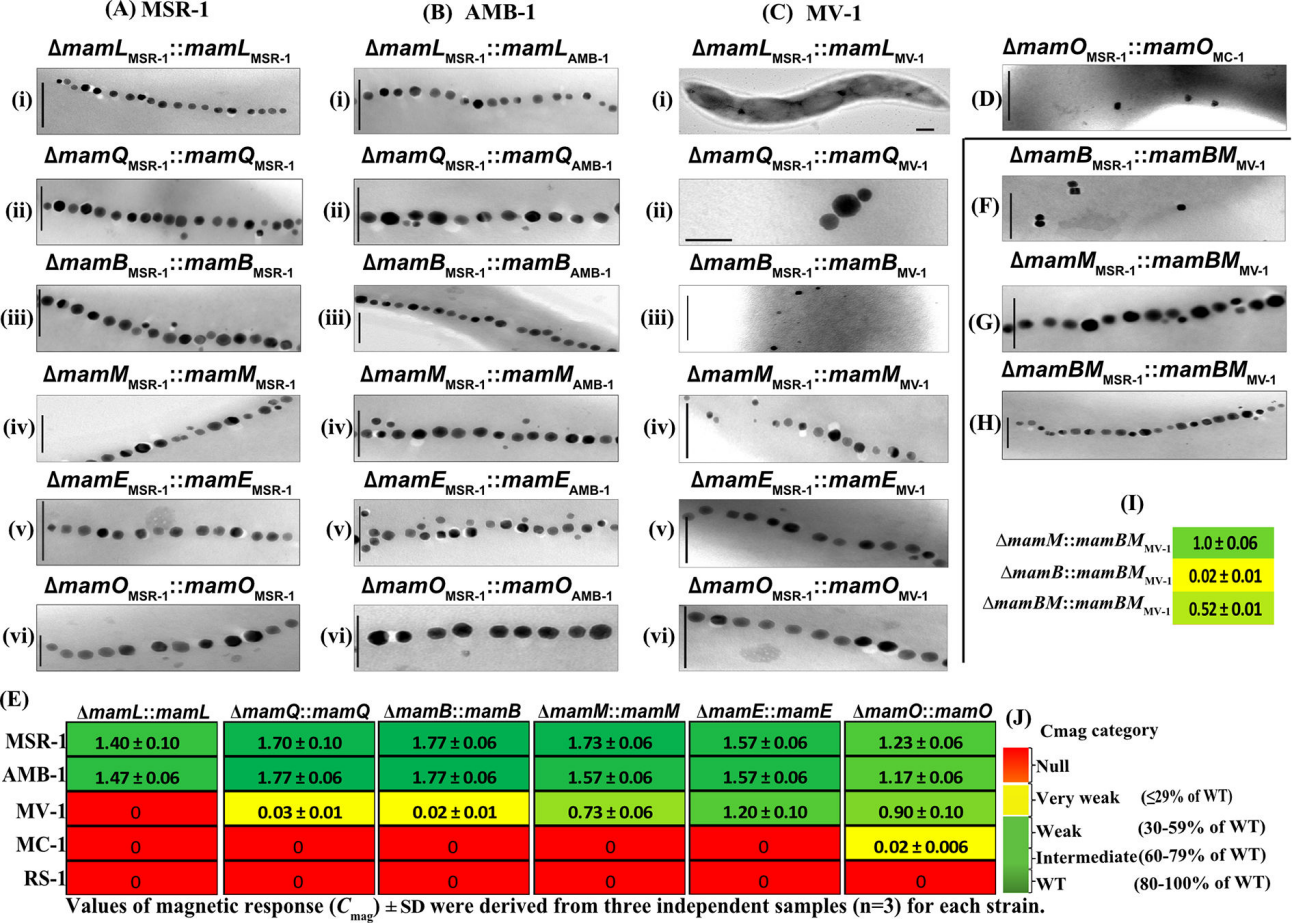
(A–C) (i–vi) TEM micrographs of representative single-gene deletion mutants (Δ*mamLQBMEO*) complemented with corresponding genes (*mamLQBMEO*) from MSR-1, AMB-1, and MV-1 respectively. (**D**) TEM micrograph of single-gene deletion mutant ∆*mamO*_MSR-1_ complemented with *mamO*_MC-1_. (**E**) Magnetic response (C_mag_) of fixed cells of the mutants depicted in micrographs. The values are derived from three independent samples (*n* = 3). (**F–H**) TEM micrographs of single-gene deletion mutants Δ*mamB*_MSR-1_, Δ*mamM*_MSR-1_, and double-deletion mutant Δ*mamBM*_MSR-1_ complemented with *mamBM* from MV-1 and C_mag_ of these mutants (**I**). (**J**) C_mag_ category. Scale bars = 200 nm.

Genes (*mamLQBMEO*) from these strains were cloned on pBAM-Tn5-P*_mamH_* and transferred by transposition into single-gene deletion MSR-1 strains (Fig. S1Ai-vi), which resulted in clones harboring single copies of introduced genes at random chromosomal locations (58). All single genes were put under the control of the moderate-strength P*_mamH_* promoter, which is known to drive transcription of the first genes of the *mamAB*op of MSR-1 (59). Orthologues from the recipient strain MSR-1 were used as a positive control, and all of them essentially restored wild-type (WT)-like magnetosome biomineralization in their respective deletion mutant as revealed by C_mag_ (magnetic response, a light-scattering-based proxy for the semiquantitative estimation of average magnetic alignment of cells [60]) and transmission electron microscopy (TEM) micrographs (Table 1; Fig. 2A). Mam proteins from more remotely related donors (MV-1, MC-1, and RS-1) were also fused to enhanced green fluorescent protein (EGFP) at either their N- or C-termini to monitor their expression and localization by fluorescence microscopy.

**TABLE 1:** Characteristics of generated mutants

Strains	Magnetic response (Cmag) category	Average magnetosome size (nm)^a^	Magnetosome size (% of WT)	No. of magnetosomes per cell^a^	Maximum size (nm)	Shape factor (SF)
WT_MSR-1_	1.70 ± 0.10	46.40 ± 11.52	100	29.2 ± 8.0	92.35	0.91
*∆mamL::*P*_mamH_-mamL*_MSR-1_	WT	30.40 ± 8.02	65.51	24.3 ± 7.2	54.12	0.91
*∆mamL::*P*_mamH_-mamL*_AMB-1_	WT	25.03 ± 7.78	55.42	28.48 ± 6.5	52.32	0.90
*∆mamL::*P*_mamH_-mamL*_MV-1_	None					
*∆mamL::*P*_mamH_-mamL*_MC-1_	None					
*∆mamL::*P*_mamH_-mamL*_RS-1_	None					
*∆mamQ::*P*_mamH-_mamQ*_MSR-1_	WT	43.07 ± 11.52	92.82	27.4 ± 10.0	73.45	0.90
*∆mamQ::* P*_mamH-_mamQ*_AMB-1_	WT	51.72 ± 13.74	111.46	29.40 ± 8.9	90.25	0.93
*∆mamQ::*P*_mamH-_mamQ*_MV-1_	Very weak	39.16 ± 12.67	84.4	3.2 ± 1.5	88.69	0.93
*∆mamQ::*P*_mamH-_mamQ*_MC-1_	None					
*∆mamQ::*P*_mamH-_mamQ*_RS-1_	None					
*∆mamE::*P*_mamH_-mamE*_MSR-1_	WT	29.95 ± 8.86	65.54	25.1 ± 6.5	58.12	0.91
*∆mamE::*P*_mamH_-mamE*_AMB-1_	WT	31.82 ± 8.11	68.57	27.16 ± 6.8	56.98	0.91
*∆mamE::*P*_mamH_-mamE*_MV-1_	Intermediate	26.30 ± 6.35	56.68	27.80 ± 4.5	43.87	0.90
*∆mamE::*P*_mamH_-mamE*_MC-1_	None					
*∆mamE::*P*_mamH_-mamE*_RS-1_	None					
*∆mamO::*P*_mamH_-mamO*_MSR-1_	Intermediate	31.88 ± 10.30	68.7	19.7 ± 6.2	58.69	0.91
*∆mamO::*P*_mamH_-mamO*_AMB-1_	Intermediate	32.84 ± 9.76	70.77	18.16 ± 4.7	59.47	0.90
*∆mamO::*P*_mamH_-mamO*_MV-1_	Weak	24.56 ± 5.94	52.93	21.40 ± 4.6	38.69	0.89
*∆mamO::*P*_mamH_-mamO*_MC-1_	Very weak	23.73 ± 7.93	51.14	6.2 ± 3.5	34.12	0.75
*∆mamO::*P*_mamH_-mamO*_RS-1_	None					
*∆mamB::*P*_mamH_-mamB*_MSR-1_	WT	37.35 ± 10.39	80.5	33.5 ± 10.3	64.56	0.92
*∆mamB::*P*_mamH_-mamB*_AMB-1_	WT	34.51 ± 10.02	74.37	30.78 ± 9.5	59.47	0.94
*∆mamB::*P*_mamH_-mamB*_MV-1_	Very weak	13.81 ± 5.16	29.76	8.9 ± 4.8	38.94	0.88
*∆mamB::*P*_mamH_-mamB*_MC-1_	None					
*∆mamB::*P*_mamH_-mamB*_RS-1_	None					
*∆mamM::*P*_mamH_-mamM*_MSR-1_	WT	40.57 ± 12.41	87.43	29.1 ± 18.8	63.56	0.90
*∆mamM::*P*_mamH_-mamM*_AMB-1_	WT	34.43 ± 10.16	74.2	25.95 ± 7.4	64.48	0.92
*∆mamM::*P*_mamH_-mamM*_MV-1_	Weak	32.16 ± 8.84	69.31	24.6 ± 7.7	48.59	0.92
*∆mamM::*P*_mamH_-mamM*_MC-1_	None					
*∆mamM::*P*_mamH_-mamM*_RS-1_	None					
*∆mamB::mamBM*_MV-1_	Very weak	25.00 ± 12.51	53.88	3.85 ± 2.35	41.25	0.84
*∆mamM::mamBM*_MV-1_	Weak	33.8 ± 10.53	72.84	18.6 ± 6.30	44.8	0.91
*∆mamBM::mamBM*_MV-1_	Weak	35.93 ± 11.30	77.43	16.75 ± 6.40	36.98	0.9
*∆mamAB::mamABop*_AMB-1_	WT	27.82 ± 11.25	59.70	27.6 ± 2.07	42.64	0.92
*∆mamAB::mamABop*_MV-1_	None					
*∆MAI*_AMB-1_*::MAG*_AMB-1_	Intermediate	46.10 ± 15.43	+10%_AMB-1_	17 ± 5.14	92.65	0.82
*∆A13-∆AB*_MSR-1_*::MAG*_AMB-1_	Intermediate	43.73 ± 12.26	94.25	18.0 ± 3.87	90.65	0.9
WT _MSR-1_*::MAG*_AMB-1_	WT	45.6 ± 13.68	98.27	65.2 ± 12.67	91.58	0.92

^a^ Values are mean ± standard deviations. C_mag_ category: WT C_mag_ (80–100% of WT), intermediate (60–79% of WT C_mag_), weak (30–59% of WT C_mag_), and very weak (less than 30% of WT C_mag_).

Foreign magnetosome genes *mamLQBMEO* were selected because they (i) are present in many, if not all, diverse magnetite-producing MTB and thus cover a wide range of sequence divergence, (ii) have essential functions in magnetite biomineralization and are associated with distinct mutant phenotypes that are easy to monitor (61); (iii) cover the structural complexity ranging from small (MamL) to large (MamEO) proteins (11, 12), and (iv) have known functional dependence on interaction and heterodimerization (MamBM) (48).

MamL is a small magnetosome protein (78 aa, 8.58 kDa in MSR-1) with two predicted integral membrane α-helices that is only present in magnetite-producing MTB (62). In *Magnetospirillum* spp., it plays a role in the early biogenesis of the magnetosome membrane (MM) (5, 61, 63). Because of the inconsistent phenotypes of ∆*mamL* mutants reported in AMB-1 (=entire absence of magnetite crystals under any conditions) (63) and MSR-1 (=tiny magnetite crystals still formed at lower growth temperature) (5, 61), we re-deleted *mamL* in MSR-1 by replacement with a short stretch consisting of only the first three codons fused in frame to the last three codons (Met X X X X Stop, X = sense codon) (Fig. S2A). Strikingly, the new ∆*mamL* mutant failed to form any electron-dense particles, even when grown at lower temperature (15°C) (Fig. S2B). In contrast, the in-frame deletion mutant used in the study by Raschdorf et al. (5) still contains a rudimentary *mamL* allele consisting of the first six codons fused in frame to its last seven codons, encoding a 13-amino acid peptide that might still exhibit some residual activity. Therefore, the new ∆*mamL* mutant strain was used in all subsequent experiments.

*MamL*_AMB-1_ in ∆*mamL*_MSR-1_::P*_mamH_-mamL*_AMB-1_ restored a single coherent chain of smaller (55% of WT_MSR-1_) magnetosomes and a WT C_mag_ (Table 1). Transfer of *mamL* from MV-1*,* MC-1, and RS-1 into ∆*mamL*_MSR-1_ failed to induce biomineralization of electron-dense particles and restore a detectable C_mag_ irrespective of the insertions site. EGFP-MamL_MSR-1_ localized as a linear signal in ∆*mamL*_MSR-1_ (Fig. S3Ai) as in (5), indicating the localization of the fusion protein in the MM. In contrast, EGFP-MamL_MV-1_ in ∆m*amL*_MSR-1_ localized in patches in the CM (Fig. S3Bi). Likewise, EGFP-MamL_MC-1_ displayed a diffuse signal in the CM (Fig. S3Ci), and EGFP-MamL_RS-1_ showed patchy localization in CM in ∆*mamL*_MSR-1_ (Fig. S3Di), suggesting expression, but the lack of proper magnetosome localization of fusion proteins.

MamQ is 272 aa long with a size of 30 kDa in MSR-1; its deletion led to empty vesicles and loss of magnetosome formation (61). It acts as one of the early landmark proteins that participate in organizing other proteins within the CM before membrane invagination (5). Complementation with *mamQ*_AMB-1_ restored magnetosome biomineralization in ∆*mamQ*_MSR-1_::P*_mamH_-mamQ*_AMB-1_ to an even higher level than in WT_MSR-1_ with particle sizes enlarged to ~52 nm (+12% of WT_MSR-1_ size) (Table 1). Crystals between 50 and 60 nm were predominant (Fig. S4B). Few larger particles up to 90 nm were also observed as in the WT_MSR-1_. Complementation with *mamQ*_MV-1_ resulted in a very weak but detectable C_mag_ (0.03) (Fig. 2E). Most of the cells contained few (~3) particles (Fig. 2Cii; Fig. S1Dii) with cubo-octahedral shape (size of ~39 nm, = 84% of WT_MSR-1_ size, SF [shape factor = ratio of width to length] of 0.93) (Table 1), which were similar to those of the host MSR-1, but not to the elongated particles of the donor. Transfer of *mamQ* from MC-1 and RS-1 into ∆*mamQ*_MSR-1_ failed to restore any detectable C_mag_ (Fig. 2E) or formation of electron-dense particles (Fig. S1Eii, Fii). Fusion proteins EGFP-MamQ_MV-1_, EGFP-MamQ_MC-1_, and EGFP-MamQ_RS-1_ localized as patches in the CM (Fig. S3Bii, Cii, Dii).

MamE (772 aa, 78.036 kDa in MSR-1) is a conserved serine protease with one transmembrane helix, a magnetochrome motif, and two PDZ domains (62). In AMB-1, MamE plays a key role in the regulation of MM growth (64), and deletion of *mamE* resulted in empty MM vesicles and abolishment of magnetite synthesis (63). In RS-1, *mamE* is split in two genes that encode two separate proteins (MamE-Nter and MamE-Cter) (15). MamO is a large protein (632 aa, 65.38 kDa in MSR-1) with eight predicted transmembrane helices (62). It contains a trypsin-like serine protease domain and acts as an upstream regulator of MamE for MM growth (64, 65). Deletion of *mamO* resulted in empty MM vesicles devoid of electron-dense crystals in AMB-1 (63).

Since in-frame deletion mutants of *mamE* and *mamO* in MSR-1 were unavailable, we here first generated in-frame deletions of these two genes. Similar to the transposon insertants of *mamE* and *mamO* described earlier (66), the newly generated ∆*mamE* (Fig. S1Av) and ∆*mamO* (Fig. S1Avi) null mutants showed no C_mag_ and the absence of any magnetosome-like electron-dense particles, which, however, could be both restored to near WT_MSR-1_ level by transfer of the respective WT alleles (Fig. 2Av, vi; Table 1).Transfer of *mamE*_AMB-1_ into ∆m*amE*_MSR-1_ restored the formation of magnetite crystals (69% of WT_MSR-1_ size) (Table 1; Fig. S6A). *MamE*_MV-1_ restored the ability in ∆*mamE*_MSR-1_::P*_mamH_-mamE*_MV-1_ to produce magnetite as indicated by an intermediate C_mag_ and the presence of small magnetosome-like crystals (57% of WT_MSR-1_ size) with an SF of 0.90 close to that of the host (Table 1). Similar as MamE_MSR-1_, EGFP-MamE_MV-1_ localized as a linear signal in ∆*mamE*_MSR-1_ (Fig. S3Bv). Transfer of *mamE* from MC-1 and *mamE-Nter-mamEO-mamE-Cter* (termed *MamE*_RS-1_ onward) from RS-1 in ∆*mamE*_MSR-1_ failed to restore C_mag_ as well as the formation of any electron-dense particles (Fig. 2E; Fig. S1Ev, Fv ). EGFP-MamE_MC-1_ and EGFP-MamE_RS-1_ showed homogeneously distributed fluorescence all over the CM (Fig. S3Cv, Dv ), suggesting its failure to interact with other proteins to the MM. This homogenous localization of the large MamE orthologs from distantly related MC-1 and RS-1 may be due to their large size and the hydrophobic regions, which may interact differently with the surrounding lipids.

*MamO*_AMB-1_ in ∆m*amO*_MSR-1_ restored an intermediate C_mag_ and the formation of magnetite crystals (Table 1). Crystals between 25 and 40 nm were predominant (Fig. S6B). *MamO*_MV-1_ restored a weak C_mag_ and the presence of magnetosomes (53% of WT_MSR-1_ size) (Table 1) with no crystals >40 nm (Fig. S6B). The magnetosomes in the mutant had an SF of 0.89, which is close to that of the host (Table 1). Similar to EGFP-MamO_MSR-1_, EGFP-MamO_MV-1_ localized predominantly in a linear fashion, akin to the characteristic magnetosome chains position in ∆*mamO*_MSR-1_ (Fig. S3Bvi), indicating its proper localization within the MM. *MamO*_MC-1_in ∆*mamO*_MSR-1_ restored a very weak but detectable C_mag_ (0.02) and the formation of small irregular magnetosomes that were not aligned in a chain (Fig. 2E and D). EGFP-MamO_MC-1_ localized as a short linear signal at mid-cell (Fig. S3Cvi), indicating its localization within the MM. By contrast, transfer of *mamO*_RS-1_ into ∆*mamO*_MSR-1_ failed to produce magnetosomes in ∆*mamO*_MSR-1_::P*_mamH_-mamO*_RS-1_, and EGFP-MamO_RS-1_ was localized all over the CM (Fig. S3Dvi).

MamB (298 aa, 31.96 kDa in MSR-1) and MamM (319 aa, 34.48 kDa in MSR-1) are cation-diffusion facilitators (11), which transport ferrous iron from the bacterial cytoplasm into the magnetosome lumen (48). In addition, MamB plays a key role in MM invagination and magnetite nucleation, possibly by recruiting other proteins (48). Deletion of *mamB* in MSR-1 resulted in a lack of magnetosome vesicles, whereas *mamM* deletion caused the loss of magnetite crystals but not MM vesicles (48).

Transfer of only *mamB*_AMB-1_ into ∆*mamB*_MSR-1_ restored a WT C_mag_ and the formation of linear magnetosome chains, which were slightly smaller (74% of WT_MSR-1_ size) in the mutant ∆*mamB*_MSR-1_::*P_mamH_-mamB*_AMB-1_ (Fig. 2B iii ; Fig. S1C iii; Table 1). *MamM*_AMB-1_ in ∆*mamM*_MSR-1_ alone also restored linear magnetosome chains of smaller sizes (74% of WT_MSR-1_) (Table 1) and a WT C_mag_. Particles >65 nm were absent (Fig. S5B). In contrast, the mutant ∆*mamM*_MSR-1_::P*_mamH_-mamM*_MSR-1_ contained larger particles up to 80 nm (Fig. S5B). Complementation of ∆*mamB*_MSR-1_ with only *mamB*_MV-1_ restored a very weak C_mag_ (0.02), and cells formed few small magnetosomes (~9 per cell, SF of 0.88, ~14 nm; 30% of WT_MSR-1_ size) with irregular morphology (Fig. 2Ciii; Fig. S1Diii; Table 1; Fig. S5A). MamB_MV-1_-EGFP showed linear localization in ∆*mamB*_MSR-1_ (Fig. S3Biii), consistent with the restoration of MM vesicles. *MamM*_MV-1_ alone restored the formation of smaller magnetosomes and a weak C_mag_ (Table 1). The magnetosomes had an SF of 0.92 similar to that of MSR-1 (Table 1). MamM_MV-1_-EGFP in ∆*mamM*_MSR-1_ localized in a straight line running through the center of the mid-cell resembling a chain-like organization (Fig. S3Biv).

Transfer of *mamB* from MC-1 and RS-1 into ∆*mamB*_MSR-1_ failed to restore a detectable C_mag_ (Fig. 2E) and the biomineralization of electron-dense particles in ∆*mamB*_MSR-1_::P*_mamH_-mamB*_MC-1_ and ∆*mamB*_MSR-1_::P*_mamH_-mamB*_RS-1_, respectively (Fig. S1Eiii, Fiii). A linear fluorescence signal was observed with MamB_MC-1_-EGFP in ∆*mamB*_MSR-1_ (Fig. S3Ciii), suggesting that MM vesicle formation was probably restored. In contrast, MamB_RS-1_-EGFP showed even fluorescence all over the CM (Fig. S3Diii), indicating its failure to specifically localize within the MM. Similar to *mamB*, *mamM* from MC-1 and RS-1 in ∆*mamM*_MSR-1_ both failed to restore a C_mag_ and the biomineralization of electron-dense particles in ∆*mamM*_MSR-1_*,* respectively (Fig. S1Eiv, Fiv). MamM_MC-1_-EGFP localized as random patches in ∆*mamM*_MSR-1_ (Fig. S3Civ), while a linear signal was observed with MamM_RS-1_-EGFP (Fig. S3Div), indicating its MM localization.

In MSR-1, MamB and MamM are known to form a heterodimer, and MamM is essential for the proteolytic stability of MamB (48, 67). Therefore, we also studied the co-expression of MamB and MamM from more remotely related donors (MV-1, MC-1, and RS-1) to ensure the proper interaction with their cognate partners. To this end, the paralogous pairs from donor strains were placed under the control of different promoters from MSR-1 (*mamB*: P*_mamG_* (68), *mamM*: P*_mamH_*) in the multi-promoter setup separated by a unique nucleotide sequence (UNS) (69, 70). We then transferred the resulting mariner transposon-based constructs into single-deletion strains, Δ*mamB* and Δ*mamM*, and into the double-deletion strain Δ*mamB*Δ*mamM* of MSR-1. Co-transfer of *mamBM*_MV-1_ into ∆*mamB*_MSR-1_ restored a detectable C_mag_ (0.02), and micrographs showed few magnetosomes-like particles (~4 per cell, SF of 0.84, ~25 nm; 54% of WT_MSR-1_ size) (Fig. 2F; Table 1). Complementation of ∆*mamM*_MSR-1_ with *mamBM*_MV- 1_ restored a high C_mag_ of 1, and the formation of magnetosomes (~19 per cell, SF of 0.91, ~34 nm; 73% of WT_MSR-1_ size) (Fig. 2G; Table 1). Transfer of *mamBM*_MV-1_ into ∆*mamBM*_MSR-1_ restored a weak C_mag_ (31% of WT_MSR-1_) (Fig. 2I) with magnetosome chains (~17 per cell, ~36 nm; 77% of WT_MSR-1_ size) (Fig. 2H; Table 1). The magnetosomes in the mutants were WT_MSR-1_-like (SF of 0.90, Table 1) but not as elongated as in the donor. In conclusion, co-transfer of *mamBM*_MV-1_ in ∆*mamB_MSR-1_* caused a more efficient complementation than transfer of *mamB*_MV-1_ alone due to the presence of its cognate interacting partner *mamM*_MV-1_. In contrast, co-expression of *mamBM*_MV-1_ in ∆*mamM_MSR-1_* showed a similar result as the transfer with only *mamM*_MV-1_. Co-transfer of *mamBM* from both distantly related MC-1 and RS-1 did not result in magnetosome formation in single- and double-deletion mutants. Overall, these results indicated that single orthologues from only the closely related donor strains AMB-1 and MV-1 can fully or partially replace the function of native magnetosome genes in MSR-1. In contrast, those from more remotely related donor strains MC-1 (with the exception of *mamO*) and RS-1 entirely failed to restore magnetosome biomineralization and proper MM localization.

### Expression of entire magnetosome biosynthetic gene clusters

The selected single magnetosome genes residing in the *mamAB*op of AMB-1 and MV-1 showed partial-to-full complementation upon transfer into MSR-1, lacking the residual genes. *MamAB*op_AMB-1_ (17,706 bp in size) shows exact synteny with *mamABop*_MSR-1_ but contains an additional gene (*mamV*) of unknown function downstream *mamU* (Fig. 1B). The gene order of *mamAB*op_MV-1_ (18,179 bp in size) is similar to MSR-1 and AMB-1, although it lacks *mamH* and *mamJ* but comprises an orthologue of the cytoskeletal *mamY* (9) as well as unknown gene downstream *mamY* (29) (Fig. 1B). Next, we wanted to test whether these operons can replace the function of *mamAB*op of MSR-1. To this end, we constructed the mariner transposon-based pTps-Kn^R^-*mamAB*op_AMB-1_ and pTps-Kn^R^-*mamAB*op_MV-1_ using Gibson Assembly (71) and transferred them into a *mamAB*op-deleted strain of MSR-1.

**Fig 3:**
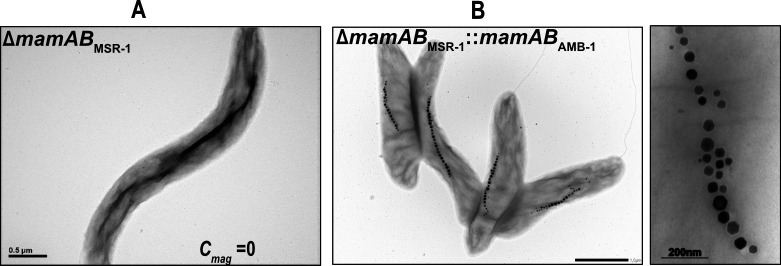
(**A**) TEM micrographs of ∆*mamABop* mutants in MSR-1. (**B**) The mutant ∆*mamAB*_MSR-1_::*mamAB*_AMB-1_ showed a linear chain of magnetosomes.

Complementation of ∆*mamAB*_MSR-1_(Fig. 3A) with *mamAB*_AMB-1_ restored a WT C_mag_ as expected, and cells of ∆*mamAB*_MSR-1_::*mamAB*_AMB-1_ showed a coherent magnetosome chains highly similar to WT_MSR-1_ (Fig. 3B; Table 1). The crystals had an SF of 0.92, thus more akin to that of the recipient MSR-1 than the donor strain AMB-1. By contrast, transfer of native *mamAB*_MV-1_ into ∆*mamAB*_MSR-1_ did not restore a C_mag_, and cells were devoid of electron-dense particles (data not shown). There are several possible reasons why the expression of *mamAB*_MV-1_ in ∆*mamAB*_MSR-1_ may have been compromised, including the possibility that MamL_MV-1_ alone is not functional, or that the native promoter from MV-1 has low activity in MSR-1, resulting in insufficient expression of MamL and other potential interactors within the *mamAB*_MV-1_. Since the *Operon-mapper* (69) algorithm predicted the existence of three putative suboperons, *mamAB*op_MV-1_ was subdivided by placing each of them under control of separate promoters from MSR-1 (P*_mamH_*_int_, P*_mamY_*, and P*_mms36_* [59]). The suboperons were fused with genes encoding fluorescent proteins mCherry, mTurquoise2, and omNeonGreen, resulting in pTps-Kn^R^-P_MSR-1_-*mamABop*_MV-1_-RG (Method S1 C). Although reporter genes (mCherry and omNeonGreen) were expressed as shown by fluorescence microscopy (Fig. S7A), transfer of pTps-Kn^R^-P_MSR-1_-*mamABop*_MV-1_-RG into ∆*mamAB_MSR-1_* failed to restore C_mag_, and cells were devoid of electron-dense particles (Fig. S7B). The lack of magnetosomes in the complemented mutant could be due to the absence of further orthologues of *mamAB*op*_Mgrpyh_*, such as *mamH,* which is known as putative iron transporter in MSR-1 (72). Therefore, we next transferred the accessory *mamDFHK*op_MV-1_ under the control of P*_mamG_* from MSR-1 into ∆*mamAB*_MSR-1_::*mamABop*_MV-1_, a strain which already harbors *mamABop*MV-1. TEM micrographs showed few irregular electron-dense particles not aligned in a chain (Fig. S7C); however, the lack of a detectable C_mag_ suggested that they did not consist of a magnetite. This indicates that *mamAB*op_MV-1_ even along with accessory genes is insufficient to confer a magnetic phenotype to MSR-1.

**Fig 4:**
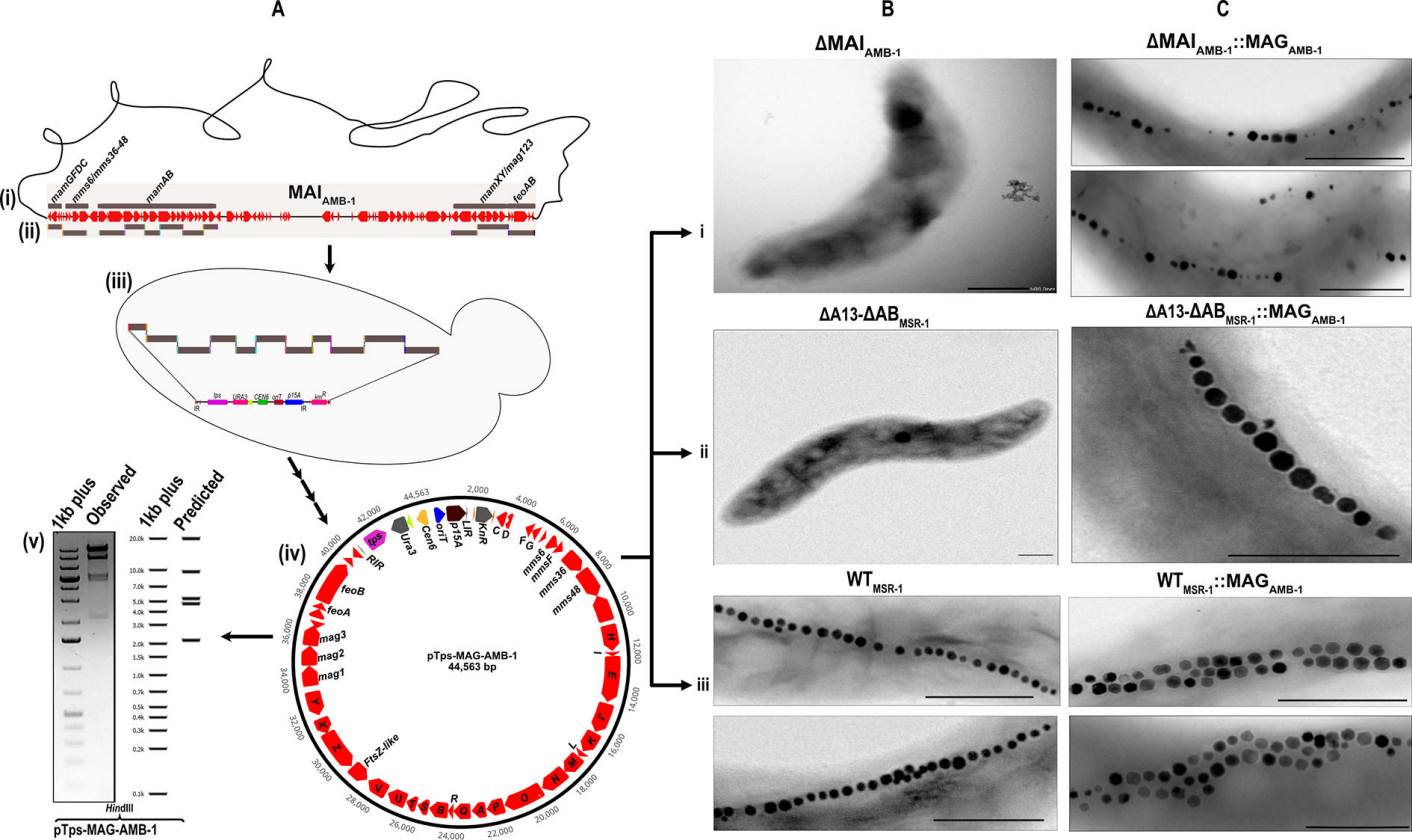
(**A, i**) Schematic representation of MAI in the genome of *M. magneticum*. (**A, ii**) The five magnetosome operons were divided into 11 fragments and transformed them along with a linearized pTps-TAR-RPA plasmid in yeast (A, iii). The final recombined plasmid (**A, iv**) was verified by restriction hydrolysis with HindIII (**A, v**). The expression cassette was transferred into ΔMAI_AMB-1_ (**B, i**), ∆A13-∆*AB*_MSR-1_ (**B, ii**), and WT_MSR-1_ (B, iii). TEM micrographs of mutant ∆MAI_AMB-1_::MAG_AMB-1_ showed a typical linear fragmented chain of magnetosomes (**C, i**). The mutant ∆A13-∆*AB*_MSR-1_:: MAG_AMB-1_ showed functional complementation with a linear chain of magnetosomes (**C, ii**). The mutant WT_MSR-1_::MAG_AMB-1_ showed a double chain of magnetosomes (C, iii).

We next attempted to replace the complete MGCs of MSR-1 by those from the foreign bacterium AMB-1 (Fig. 4Ai). To reassemble its MGC, we used transformation-associated recombination (TAR) cloning that exploits homologous recombination in yeast to assemble large DNA molecules (73, 74). To this end, the five magnetosome operons (*mamAB*, *mamGFDC*, *mms6/mms36-48*, *mamXY/mag123*, and *feoAB* operons) of AMB-1 were divided into 11 fragments between 2.3 and 5 kb, with 60 bp overlapping homologous sequences to all adjacent cluster fragments (Fig. 4Aii). These fragments were assembled and cloned into the transposable shuttle vector pTps-TAR-RPA that we had customized for replication and selection in both yeast and MSR-1 as explained in the Materials and Methods section. This successfully yielded pTps-MAG_AMB-1_, which comprised the entire MGCs as a single contiguous construct of 44.5 kb. To test its functionality, we first transferred pTps-MAG_AMB-1_ into its native MAI-deleted AMB-1 background strain ∆MAI_AMB-1_ (M. Dziuba, unpublished) (Fig. 4Bi), which yielded strain ∆MAI_AMB-1_::MAG_AMB-1_. As expected, the trans-complemented strain showed a significant C_mag_ with a fragmented magnetosome chains characteristic of AMB-1 (Fig. 4Ci; Table 1), thus proving its functionality. We next transferred pTps-MAG_AMB-1_ into ∆A13-∆*AB*_MSR-1_, in which *mamAB* was deleted in ∆A13 (accessory operons *mms6*, *mamGFDC*, and *mamXY* already co-deleted [75]) (Fig. 4Bii). Strikingly, the trans-complemented ∆A13-∆*AB*_MSR-1_::MAG_AMB-1_ exhibited a significant C_mag_ (Table 1), and TEM micrographs showed a coherent magnetosome chains of about ~18 particles as typical for the recipient strain MSR-1, instead of the fragmented magnetosome chains of the donor (Fig. 4Cii). The average size of magnetosomes was ~44 nm (94% of WT_MSR-1_ size) with an SF of 0.90 (Table 1), thus again akin to that of recipient strain.

We also tested whether the entire MGC clusters from AMB-1 and MSR-1 can be functionally co-expressed. To this end, we transferred pTps-MAG_AMB-1_ into WT_MSR-1_ (Fig. 4Biii), which already harbors its own functional version of MGC. TEM micrographs of the mutant WT_MSR-1_::MAG_AMB-1_ showed a double chain of magnetosomes in >95% of cells (Fig. 4Ciii). C_mag_ was 1.53 (90% of WT_MSR-1_), and cells contained about ~65 magnetosomes with an average size of ~45 nm (98% of WT_MSR-1_ size) (Table 1). The magnetosomes had an average SF of 0.92, which again is closer to that of MSR-1 (Table 1) than of AMB-1 (0.78). Thus, the insertion of an additional magnetosome gene cassette from AMB-1 in WT_MSR-1_ resulted in magnetosome overproduction with 2.2-fold increased magnetosome numbers as previously observed in an MSR-1 strain in which the entire MGC swas duplicated (58). However, in contrast to the homogenous MGC-duplicated strain in (58), in WT_MSR-1_::MAG_AMB-1_, the crystal size was not enlarged but remained similar to WT_MSR-1_.

## DISCUSSION

In this study, we systematically investigated the expression of foreign magnetosome genes in MSR-1. Although genetic transfer of magnetosome biosynthesis from MSR-1 to other, hitherto nonmagnetic bacteria have been accomplished, the resulting magnetosomes were lacking the structural perfection of magnetosomes in native MTB (76 - 78). Thus, MSR-1 is the preferred surrogate host for functional reconstitution of magnetosome biosynthesis pathways from foreign MTB since it can be assumed to contain the full complement of auxiliary genes needed for magnetite biomineralization (79). The genomic insertion of single copies of *mam* orthologues and gene clusters ensured expression similar to native levels and avoided detrimental effects of multi-copy expression from plasmids, which proved impractical for the expression of MGCs owing to their instability (58, 76). Regardless of the random insertion of foreign orthologues into the host chromosome via transposition, we found no difference in expression efficiency.

Single orthologues from the *Alphaproteobacteria* AMB-1 and MV-1 did restore magnetosome biosynthesis to different degrees and were properly localized, while orthologues from distantly related MC-1 (with the exception of *mamO*) and RS-1 did not. However, they were expressed at significant levels as indicated by the fluorescence of the reporter EGFP, although we cannot entirely rule out the possibility that the C-terminal part of the EGFP-Mam fusion protein was somewhat less stable or translated less efficiently. The ability of foreign orthologues to functionally substitute MSR-1 genes thus seems to be correlated with their phylogenetic distance and sequence similarities of proteins, which is between 87% and 99% (76%–95% identity) for AMB-1, 63% and 85% (29%–53% identity) for MV-1, 57% and 77% (29%–42%) for MC-1, and 38% and 71% (15%–30%) for RS-1, as compared with MSR-1 (Fig. 1C). Contrary to previous work (48), MamB_MV-1_ restored weak biomineralization in ∆*mamB*_MSR-1_, possibly due to chromosomal expression used here compared with the medium-copy number plasmid used by Uebe et al. (48) that potentially compromised expression levels in MSR-1. Orthologues from more remotely related donor strains MC-1 and RS-1 entirely failed to induce magnetosome biomineralization, except for the weak complementation by MamO_MC-1_. Despite the relatively similar protein identities of MV-1 (29%–53%) and MC-1 (29%–42%) orthologues with MSR-1 proteins, their respective activity or stability could still differ, which can affect their ability to restore magnetosome formation in the deletion mutants. The congruent topologies of the phylogenetic tree based on 16S rRNA and concatenated magnetosome proteins (80, 81) suggest the co-evolution of magnetosome proteins. The examples studied here are thought to be engaged in numerous interactions within the multiprotein-MM (14) and thus they likely require the reciprocal conservation of interaction sites that might be absent in MSR-1. Thus, the failure of complementation could be due to their inability to interact with other proteins from the unrelated host. In fact, transfer of interacting cognate partners (*mamBM*) from MV-1 increased functional complementation in *∆mam*BM_MSR-1_, while in contrast, *mamB*_MV-1_ alone only caused formation of few electron-dense particles in ∆mam*B*_MSR-1_, substantiating the need of conserved cognate interacting partners even within a rather close phylogenetic range. We, therefore, attempted the co-expression of putative interactors by transferring the core *mamAB*op, and even entire MGCs from AMB-1 and MV-1 into MSR-1. While *mamAB*op of AMB-1 could fully restore magnetosome formation in ∆*mamAB*op, the native *mamAB*op from MV-1 failed to restore biomineralization, even when complemented by the accessory *mamDFHK*op_MV-1._ Likewise, replacement of native promoters within *mamAB*op_MV-1_ by those from MSR-1 did not alleviate this problem. Future approaches, therefore, should aim for the assembly of complete MGCs, fine-tuning of expression by adjustable promoters to mimic their known transcriptional complexity, and its verification by RNA-seq (59, 82). Another potential reason for the lack of functionality might also be the absence of the native physico-chemical context (e.g., intracellular redox control) specifically required for magnetite biomineralization in the donor strains. Thus, the expression of MGC in a foreign host might also require the modification of some intracellular environmental parameters to conserve the biomineralization of particles similar in size, shape, and number as in the donor.

However, we succeeded in the assembly of a compact, portable, and fully functional version of the entire MGCs of AMB-1. This was possible by genetic modification of a shuttle vector for yeast-based TAR cloning and transferred into MSR-1, thus extending this powerful platform to MTB. TAR cloning proved superior over Gibson assembly for large MGCs, as the efficiency of the latter drastically decreases with the number of inserts and by the error-prone addition of homologous sequences (83). The construct comprising the entire MGCs of AMB-1 on pTps-MAG_AMB-1_ restored the ability to biomineralize magnetite both in MGC deletion mutants of the native donor AMB-1 and MSR-1. This provides proof of principle that entire MGCs can be transplanted between different foreign MTB and fully substitute their functions. Furthermore, the transfer of MGC from AMB-1 into WT_MSR-1_ generated a strain with a doubled, yet distinct set of magnetosome genes, which resulted in the overproduction of magnetosomes with a reduced risk of homologous recombination between the two divergent MGC versions.

Intriguingly, while the transfer of MGC from AMB-1 into ∆MAI_AMB-1_ strain restored its characteristic fragmented magnetosome chains with slightly elongated crystals (SF of 0.82), magnetosomes formed in MSR-1 deletion mutants and WT_MSR-1_ upon transfer of pTps-MAG_AMB-1_ were essentially identical to those in WT_MSR-1_, with a nearly isotropic shape (SF of 0.9–0.92) and the characteristic tightly spaced, gap-free magnetosome chains organization. This might be partially explained by the absence of further genes in MSR-1, such as the genomic islet outside the MAI of AMB-1 that was recently reported to be associated with the gapped-magnetosome chains phenotype (84). Similarly, in all MSR-1 mutants in which single genes from MV-1 restored magnetosome biomineralization, the crystals were either aberrantly shaped, or more similar to the isotropic cubo-octahedral crystals of MSR-1 rather than the elongated crystals of MV-1, even if some individual crystals appeared slightly elongated, as it is sometimes observed in WT_MSR-1_. The absence of morphogenetic effects suggests that transferred genes from MV-1 and AMB-1 alone are not involved in, or sufficient for the formation of distinct shapes, but morphogenesis of crystals is probably controlled by other unknown determinants inside or outside the MAI of respective donors.

In conclusion, this study provides the first proof of principle that MSR-1 is a suitable surrogate host for the functional expression of magnetosome genes from foreign MTB. However, the expression of genes from more remotely donors will likely require the assembly, engineering, and transfer of larger gene sets or entire MGCs. This would be highly attractive for reconfiguration and engineering of MSR-1 for the biomineralization of differently shaped magnetite crystals with fine-tuned magnetic properties that would be of high value in biotechnical and biomedical applications.

## MATERIALS AND METHODS

### Bacterial strains and culture conditions

MSR-1 was cultivated micro-aerobically in modified flask standard medium (FSM) (85) at 28°C and 120 rpm agitation, if not mentioned otherwise. *Escherichia coli* was grown in lysogeny broth at 37°C and shaking at 180 rpm. Donor strain *E. coli* WM3064 (W. Metcalf, unpublished) was cultivated with 0.1 mM DL*-a,*ε-diaminopimelic acid. Selection of clones and transconjugants was carried out on agar-solidified media (1.5% (wt/vol)] by the addition of kanamycin/chloramphenicol at concentrations of 25 µg/mL (*E. coli*) and 5 µg/mL (MSR-1). Optical densities (ODs) were determined photometrically at 565 nm for MSR-1 strains, and 600 nm for *E. coli*. The coefficient of magnetically induced differential light scattering (C_mag_, magnetic response) was determined as reported earlier (60). *Saccharomyces cerevisiae* BY4741 was used for TAR cloning. Cultivations were performed at 30°C in yeast extract peptone dextrose (YPD) medium (20 g L^−1^ glucose, 10 g L^−1^ peptone, 10 g L^−1^ yeast extract, and pH 6.5). Selection medium (20 g L^−1^ glucose, 7 g L^−1^ yeast nitrogen base without amino acids, 2 g L^−1^ amino acid [L-histidine, L-Leucine, and L-Methionine] mix without uracil, and pH 6.5] was used for the selection of transformants. Bacterial strains used in this study are listed in (Table S1A).

### Molecular and genetic techniques

Oligonucleotides (Table S1B) were purchased from Sigma-Aldrich (Steinheim, Germany). Plasmids were constructed by standard recombinant techniques as described below. All constructs and selected amplicons from the mutants were sequenced by Macrogen Europe (Amsterdam, the Netherlands). The plasmids used and generated in this study are listed in Table S1C. DNA synthesis of genes from MC-1 was carried out by ATG:biosynthetics GmbH. Sequence-verified DNA fragments were delivered in pGH standard vector harboring an *ampR* (bla) gene for selection on ampicillin. Genes from MSR-1, AMB-1, MV-1, and RS-1 were amplified by PCR from the respective genomic DNA. Strain AMB-1 was grown in the enriched magnetic spirillum growth medium (86), strain MV-1 was grown anaerobically with sodium succinate as electron donor and nitrous oxide as terminal electron acceptor (54), and strain RS-1 was grown anaerobically using sodium pyruvate as electron donor and sodium fumarate as terminal electron acceptor (37).

### Bioinformatic analyses

The phylogenetic tree of 16S rRNA gene sequences of selected MTB was constructed using the neighbor-joining method (87) and Jukes-Cantor correction (88) by applying 1,000 bootstrap resamplings (89). All these programs are available in the Geneious Prime software (https://www.geneious.com/).

### Construction of markerless site-specific deletions

Markerless in-frame deletion of *mamL*, *mamE*, *mamO*, and *mamAB*op in ∆A13 in MSR-1 was conducted using RecA-mediated homologous recombination based on counterselection systems described previously (42). For the construction of deletion plasmids, homologous regions of ca 1–1.5 kb up- and downstream regions of *mamL*, *mamE*, *mamO*, and *mamAB*op were amplified using a proofreading DNA polymerase, fused by an overlapping extension PCR. The PCR products were purified from the agarose gel using a gel extraction kit (Zymo Research, Irvine, CA, USA) and cloned into pORFM digested with SalI and NotI by Gibson assembly (71). The plasmids were isolated from the correct clones using a Zymo Research kit and sequenced by Macrogen Europe (Amsterdam, the Netherlands).

### Construction of trans-complementation vectors

To trans-complement ∆gene strains of MSR-1, using the expression level of foreign genes close to the recipient level, we cloned a PCR fragment encompassing the respective gene into a Tn5-based insertion vector (Method S1A) with promoter from MSR-1. Transfer of the resulting constructs into MSR-1 via conjugation resulted in the random insertion of the expression cassette into the chromosome of the host. To study the localization of magnetosome proteins from distantly donor strains, the genes were fused with e*gfp* and cloned in pBam-Tn5 plasmid (Method S1A). For the co-expression of paralogues (*mamB* and *mamM*) from remotely related donor strains (MV-1, MC-1, and RS-1), these genes were placed under the control of P*_mamG_* and P*_mamH_* from MSR-1 separated by UNS and were cloned into pTps-Kn^R^-RPA (accession no. OP837537.1; Method S1B ) by Gibson assembly. To trans-complement *mamAB*op from AMB-1 and MV-,1 *MamAB*op was amplified into four to five fragments with primers having complementary overhangs ranging from 3 to 4 kb and cloned into linearized pTps-Kn^R^-RPA (Method S1B) using Gibson assembly. Similarly, *mamDFHK*op from MV-1 was cloned under control of P*_mamG_* promoter from MSR-1 into pTps-Cm^R^-RPA (accession no. OP837538.1; Method S1B) containing chloramphenicol resistance gene using Gibson assembly. *MamAB*op_MV-1_ was further subdivided into three suboperons and fused each suboperon with mCherry, mTurquoise2, and omNeonGreen, resulting in pTps-RPA-P_MSR-1_-*mamAB*op_MV-1_-RG (Method S1C). All the constructs were verified by Sanger sequencing. The sequencing result of pTps-RPA-P_MSR-1_-*mamAB*op_MV-1_-RG showed point mutation at the C-terimus of *mamA* resulting in frameshift in mTurquoise2. Although mTurquoise2 would be unfunctional, this construct was transferred into MSR-1.

### Construction of large constructs using TAR cloning

To carry out the TAR cloning, we constructed a compatible shuttle vector (pTps-TAR-RPA [accession no. OP837536.1], Method S1D), which harbors a MycoMar transposase gene (*tps*) (90), inverted repeats (IRs) (pFNLTP16 H3 [accession no. DQ236098]), origin of transfer *oriT* (*Pseudomonas aeruginosa* plasmid Birmingham IncP-alpha [L27758]), origin of replication (*p15A*) (pACYC177 [X06402] [91]), CEN6/ARS4 (centromere sequence and autonomously replicating sequence) (pRS415 [U03449], a uracil auxotroph marker (pRS416 [U03450]) for yeast selection, and a kanamycin-resistance cassette (pACYC177 [X06402] [91]) for selection in magnetospirilla recipients. For TAR cloning, *S. cerevisiae* BY4741 was cultivated in liquid YPD medium to OD_600_ 1.2. The culture was harvested at room temperature (RT) and washed with ½ volume of ddH_2_O. The cells were finally resuspended in ddH_2_O (1/50 volume of the culture). One transformation mixture contained 100 µL of the resuspended cells (~1 × 10^8^) and 360 µL transformation mix containing 240 µL of 50% (wt/vol) PEG4000, 36 µL lithium acetate, 50 µL sheared salmon sperm DNA (2 mg/mL, boiled for 5 min at 99°C), and 34 µL of DNA fragments in an equimolar amount in ddH_2_O. The transformation mixture was incubated for 50 min at 42°C. After centrifugation, the supernatant was removed, and the cells were resuspended in 1,000 µL ddH_2_O, and 200 µL was plated/streaked on solid selection medium. After 3–5 d, selected clones were cultivated in liquid selection medium, and plasmid DNA was isolated using a modified alkaline lysis protocol: 10 mL of the selected clone cultivated in the selection medium was harvested and resuspended in 200 µL resuspension buffer containing 1 M sorbitol and 5 U/µL zymolyase, pH 7.5. The suspension was incubated for 60 min at 30°C, and spheroplasts were harvested. Subsequently, the standard alkaline lysis protocol was carried out. The isolated plasmid DNA from *S. cerevisiae* BY4741 was transformed into *E. coli* Neb10ß. Clones harboring the correct plasmids were verified by PCR amplification of the fragments and restriction hydrolysis of plasmid DNA isolated using the standard alkaline lysis. The verified plasmids were transformed into donor *E. coli* WM3064 before conjugation.

### Conjugation

Plasmid transfer by biparental conjugation was performed with donor strain *E. coli* WM3064 consisting of the verified construct and MSR-1 strains as the acceptor strain as described previously (40). In-frame markerless chromosomal deletion was generated following the conjugative transfer of the plasmid to MSR-1 and homologous recombination utilizing GalK-based counterselection as previously described (42). Successful deletions and insertions yielded deletion strains (Table S1A) and trans-conjugants strains (Table 1), respectively.

### Screening of transconjugants and PCR-test for cassette integrity

The transconjugants were transferred into 96-well plates with 150 µL of FSM containing the appropriate antibiotic concentration. The mutants were screened for integration of the expression cassette by PCR using primer pairs (Table S1B). In the case of large expression cassettes, numerous primer pairs covering the transferred cassette were used to ascertain the integrity of the transferred operons in the mutants.

### Fluorescence microscopy

For localization studies of the EGFP fusion proteins, Structured Illumination Fluorescent microscopy (SIM) was performed on an Eclipse Ti2-E N-SIM E fluorescence microscope (Nikon) equipped with a CFI SR Apo TIRF AC 100× H NA1.49 Oil objective lens, a hardware based “perfect focus system” and an Orca Flash4.0 LT Plus sCMOS camera (Hamamatsu). Sample preparation, fluorescence excitation with 488 for imaging GFP and image reconstruction and analysis were performed as reported previously (92).

### Transmission electron microscopy

For TEM analysis, the strains were cultivated under anoxic conditions in FSM at 24°C for 48 h, fixed in formaldehyde (1.8%), adsorbed onto carbon-coated copper grids (F200-CU carbon support film, 200 mesh; Electron Microscopy Sciences, Hatfield, UK), and washed three times with double-distilled water (ddH_2_O). TEM was performed on a JEM-2100 instrument (JEOL Ltd., Tokyo, Japan) at 80 kV. Images were captured with a Gatan model 782 ES500W Erlangshen charge-coupled device camera (Gatan Inc., Pleasanton, CA, USA) with the software Digital Micrograph 1.80.70 (Gatan Inc.). For data analysis and measurements, the software ImageJ Fiji V1.50c (93) was used.

## SUPPLEMENTAL MATERIAL

Supplemental text mbio.03282-22-s0001.docx Supplemental methods.

Fig. S1 mbio.03282-22-s0002.tif Supplemental TEM micrographs of mutants.

Fig. S2 mbio.03282-22-s0003.tif Schematic comparison of the remnant of *mamL* within the *mamAB*op in the deletion mutants of MSR-1.

Fig. S3 mbio.03282-22-s0004.tif SIM micrographs.

Fig. S4 mbio.03282-22-s0005.tif Magnetosome distribution in the mutants with *mamL* and *mamQ.*

Fig. S5 mbio.03282-22-s0006.tif Magnetosome size distribution in the mutants with *mamB* and *ammM*.

Fig. S6 mbio.03282-22-s0007.tif Magnetosome size distribution in the mutants with *mamE* and *mamO*.

Fig. S7 mbio.03282-22-s0008.tif Mutants with operons from MV-1.

Table S1 mbio.03282-22-s0009.docx Strains, primers, and plasmids used in this study.

ASM does not own the copyrights to Supplemental Material that may be linked to, or accessed through, an article. The authors have granted ASM a non-exclusive, world-wide license to publish the Supplemental Material files. Please contact the corresponding author directly for reuse.
