# Supplementary material for: Functional expression of foreign magnetosome genes in the alphaproteobacterium Magnetospirillum gryphiswaldense
Source: mBio. 2023 Jun 15;14(4):e03282-22. doi: 10.1128/mbio.03282-22 (PMC10470508; doi:10.1128/mbio.03282-22)
Supplement: Supplemental text — Supplemental methods. [file mbio.03282-22-s0001.docx]

**Supplementary Methods**

**Method S1 A. Construction of Tn5 transposon-based plasmids**

pBAM-Tn5-P*_mamDC_*_45_-*mamC-egfp* (1) was modified into pBAM-Tn5-P*_mamDC_*_45_ with *N*-terminus *egfp*/*C*-terminus *egfp.* The construct P*_mamDC_*_45_-*mamC*_MSR-1_ was digested with Ndel and BamHI and ligated with *egfp*-HL (HL=helix, for *N*-terminal gene fusions) and HL-*egfp* (for *C*-terminal gene fusion), both amplified from pBam-Tn5-Ptet-*popZ*-HL-*egfp* (Pfeiffer et al. 2019), digested with same restriction enzymes. The resulting constructs pBAM-Tn5-P*_mamDC_*_45_-*egfp*-HL and pBAM-Tn5-P *_mamDC_*_45_-HL-*egfp* were used for *N*-terminal/*C*-terminal reporter fusion constructs respectively. To transfer single genes, the promoter P*_mamDC_*_45_ in the construct pBAM-Tn5-P *_mamDC_*_45_-HL-*egfp* was replaced with a weaker promoter P*_mamH_*. To replace the promoter, the plasmid was digested with XhoI and HindIII and fused with P*_mamH_*, amplified by PCR from gDNA from MSR-1, digested with same enzymes, resulting in pBAM-Tn5-P*_mamH_*-HL-*egfp*. This generated construct was used to generate all constructs containing single gene without a reporter gene *egfp*. For this purpose, the plasmid pBAM-Tn5-P*_mamH_*-HL-*egfp* was digested with NdeI and BamHI, and ligated with amplified genes by PCR from respective genomic materials digested with same restriction enzymes. For fusion constructs, a stronger promoter P*_mam_*_DC_ was used to get sufficient expression and proper localization of fluorescence signals of these proteins. For the *N*-terminal gene fusion construct, pBAM-Tn5-P*_mamDC_*_45_-*egfp*-HL was digested with EcoRV and BamHI or only with EcoRV and dephosphorylated. The linearized plasmid was ligated with amplified genes (*mamL, Q, E, O* from strains included in this study) digested with same restriction enzymes. For the *C*-terminal gene fusion construct, pBAM-Tn5-P *_mamDC_*_45_-HL-*egfp* was digested with Ndel and SalI and ligated to amplified genes (*mamB, M*) digested with same restriction enzymes.

**Method S1 B. Construction of MycoMar based plasmids pTps-Kn^R^-RPA and pTps-Cm^R^-RPA**

MycoMar transposable element (2) has been used in Gram-negative hosts for genetic modification. pTps-Kn^R^-RPA was constructed by modification of pTps-*XYZ* (3). pTps-XYZ contained a vector backbone (*p15A*-*tps*-*oriT*-Gm^R^) harboring *p15A* origin of replication, MycoMar transposase gene (*tps*), origin of transfer (*oriT*), inverted repeats (IR), gentamycin cassette (Gm^R^). and mam*XYZ*op from MSR-1. A gentamycin cassette (Gm^R^) and *mamXY*op in the construct pTps-*XYZ* were replaced with a kanamycin cassette (Kn^R^) and unique nucleotide sequences (UNS1-multiple cloning sites (MCS)-UNSX) amplified from pBamII-Tn7 (Renè Uebe, unpublished) with standard digestion (with DraIII and HindIII) and ligation reaction resulting in pTps-Kn^R^-RPA. The construct pTps-Kn^R^-RPA was verified by sequencing. pTps-Kn^R^-RPA was used to construct pTps-Kn^R^-*mamAB*op_AMB-1_ and pTps-Kn^R^-*mamAB*op_MV-1_ containing *mamAB*op from AMB-1 and MV-1. A kanamycin cassette in pTps-Kn^R^-RPA was replaced with a chloramphenicol cassette using the Red/ET recombination system, resulting in pTps-Cm^R^-RPA. The construct pTps-Cm^R^-RPA was also verified by sequencing. pTps-Cm^R^-RPA was used to construct pTps-Cm^R^-P*_mamG-_mamDFHK*op_MV-1_. The sequences of pTps-Kn^R^-RPA and pTps-Cm^R^-RPA were deposited in GenBank under the accession numbers (OP837537) and (OP837538) respectively.

**Method S1 C. Construction of plasmid with sub-divided *mamAB*op_MV-1_**

Based on transcriptional complexity of *mamAB*op in MSR-1 (4), *mamAB*op from MV-1 was analysed using a web server *Operon-mapper* (Taboada et al. 2018), which predicted the existence of three putative sub-operons (*mamIEKL*-*MNOPA*-*QRBST*HP). Genes encoding fluorescent proteins mCherry, mTurquoise2 were fused to the last genes of the first two operons (*mamL* and *mamA*, respectively), and omNeonGreen was fused to *mamB* in the third operon as reporters for the expression of fusion proteins. Each putative operon was fused with reporter genes was placed under the control of promoters from MSR-1 [P_mamHint_, P_mamY_, P_mms36_  (4)] as depicted below.


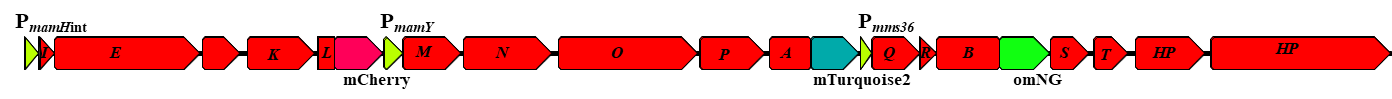


The expression cassette was fragmented into 11 fragments, which later on were reduced to 4 by overlap extension PCR and assembled into linearized pTps-Kn^R^-RPA plasmid by Gibson Assembly resulting in pTps-Kn^R^-P _MSR-1_- mamABop_MV-1_-RG.

**Method S1 D. Construction of a shuttle vector pTps-TAR-RPA**

MycoMar based pTps-Kn^R^-RPA plasmid was modified into a shuttle vector suitable for yeast, standard cloning procedures in *E. coli*, and transformation and integration of the gene cluster into MSR-1 genome. CEN6/ARS4 ori for replication in yeast and URA3 encoding orotidine 5’-phosphate decarboxylase for counter-selection in yeast were amplified from pAG416Gal-ccdB (5) and inserted in-between *oriT-tps* into pTps-Kn^R^-RPA plasmid by standard digestion/ligation method, resulting in pTPs-TAR-RPA plasmid that contains *oriT-p15A-IR-KnR-UNS1-MCS-UNSX-IR-tps-Cen6/Ura3*. The inserted components were verified by sanger sequencing. The sequences of this plasmid was deposited in GenBank under the accession number: pTps-TAR-RPA (OP837536).

References

1. Borg, Sarah; Hofmann, Julia; Pollithy, Anna; Lang, Claus; Schüler, Dirk (2014): New vectors for chromosomal integration enable high-level constitutive or inducible magnetosome expression of fusion proteins in Magnetospirillum gryphiswaldense. In: Applied and environmental microbiology 80 (8), S. 2609–2616. DOI: 10.1128/AEM.00192-14.
2. Rubin, E. J.; Akerley, B. J.; Novik, V. N.; Lampe, D. J.; Husson, R. N.; Mekalanos, J. J. (1999): In vivo transposition of mariner-based elements in enteric bacteria and mycobacteria. In: Proc. Natl. Acad. Sci. U.S.A. 96 (4), S. 1645–1650. DOI: 10.1073/pnas.96.4.1645.
3. Kolinko, Isabel; Lohße, Anna; Borg, Sarah; Raschdorf, Oliver; Jogler, Christian; Tu, Qiang et al. (2014): Biosynthesis of magnetic nanostructures in a foreign organism by transfer of bacterial magnetosome gene clusters. In: Nature nanotechnology 9 (3), S. 193–197. DOI: 10.1038/NNANO.2014.13.
4. Dziuba, Marina; Riese, Cornelius N.; Borgert, Lion; Wittchen, Manuel; Busche, Tobias; Kalinowski, Jörn et al. (2021): The Complex Transcriptional Landscape of Magnetosome Gene Clusters in Magnetospirillum gryphiswaldense. In: mSystems 6 (5), e0089321. DOI: 10.1128/mSystems.00893-21.
5. Alberti, Simon; Gitler, Aaron D.; Lindquist, Susan (2007): A suite of Gateway cloning vectors for high-throughput genetic analysis in Saccharomyces cerevisiae. In: Yeast 24 (10), S. 913–919. DOI: 10.1002/yea.1502.
