# Supplementary figures and images for: Functional expression of foreign magnetosome genes in the alphaproteobacterium Magnetospirillum gryphiswaldense
Source: mBio. 2023 Jun 15;14(4):e03282-22. doi: 10.1128/mbio.03282-22 (PMC10470508; doi:10.1128/mbio.03282-22)

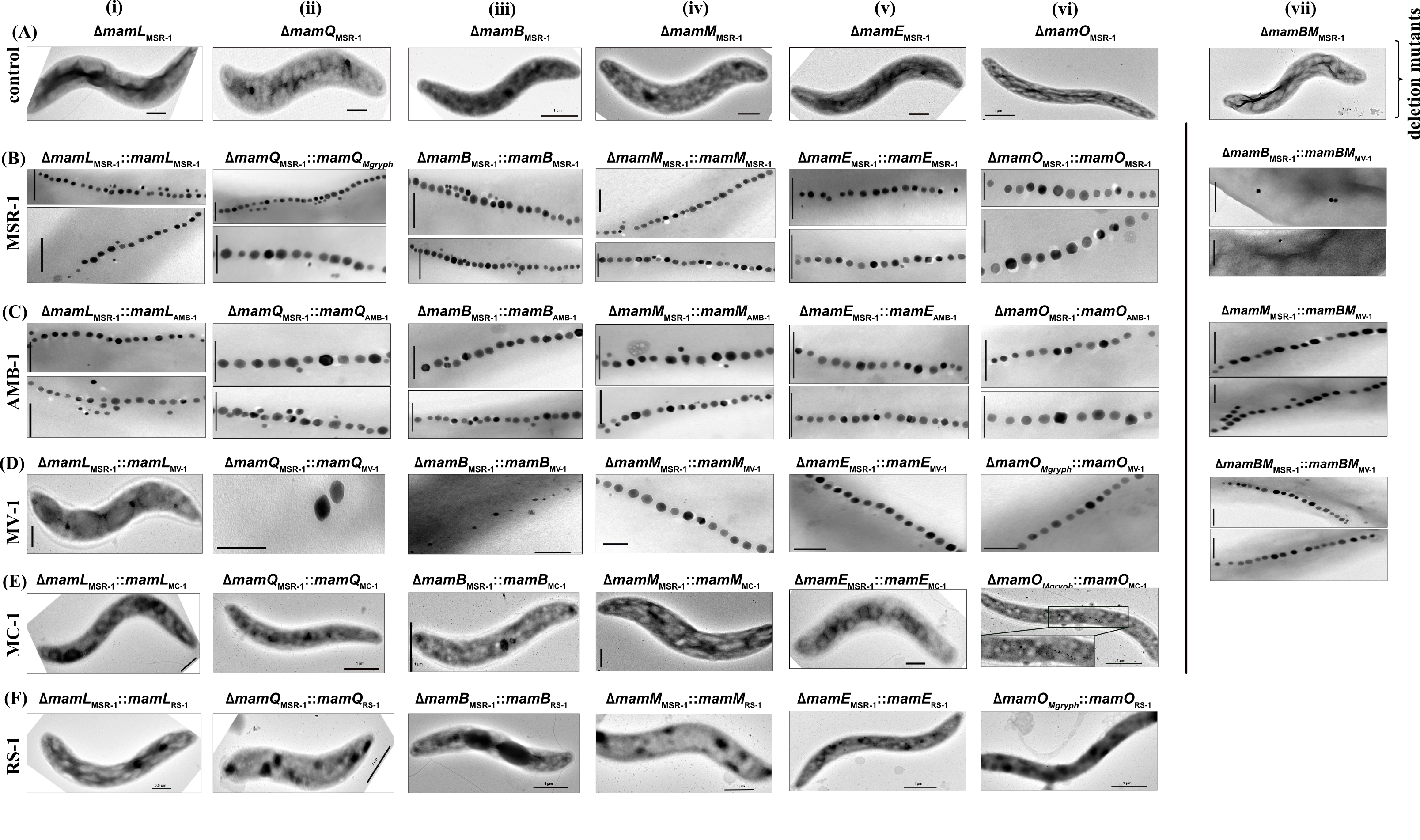

Supplement: Fig. S1 — Supplemental TEM micrographs of mutants. [file mbio.03282-22-s0002.tif]

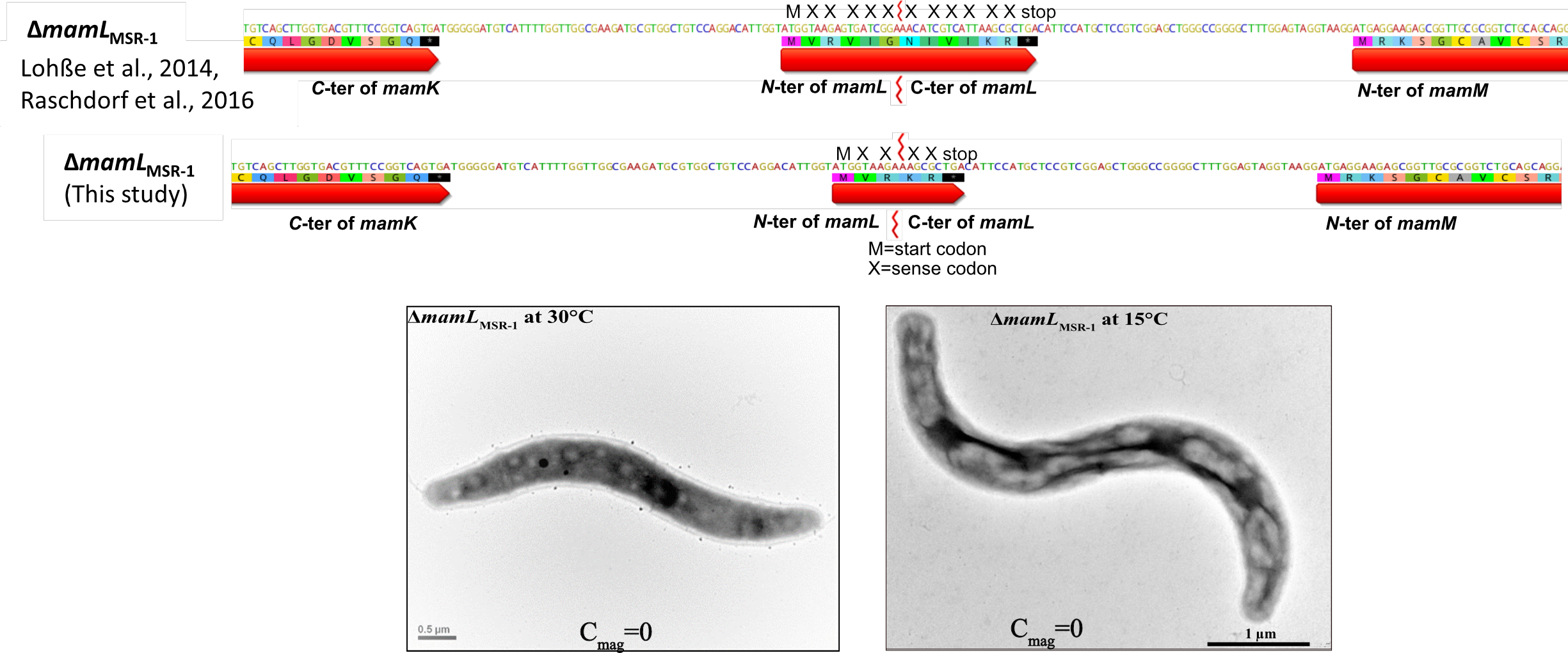

Supplement: Fig. S2 — Schematic comparison of the remnant of mamL within the mamABop in the deletion mutants of MSR-1. [file mbio.03282-22-s0003.tif]

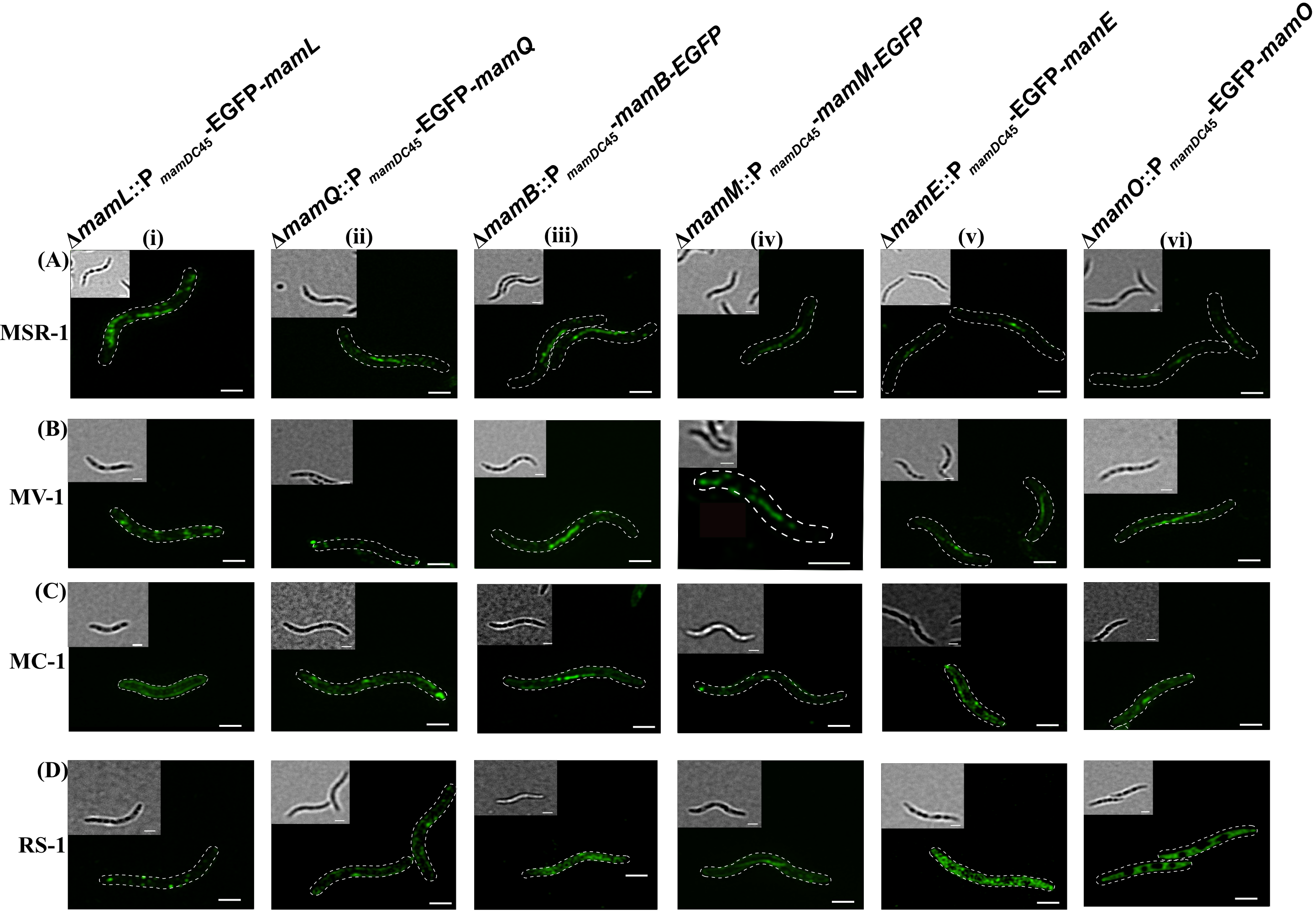

Supplement: Fig. S3 — SIM micrographs. [file mbio.03282-22-s0004.tif]

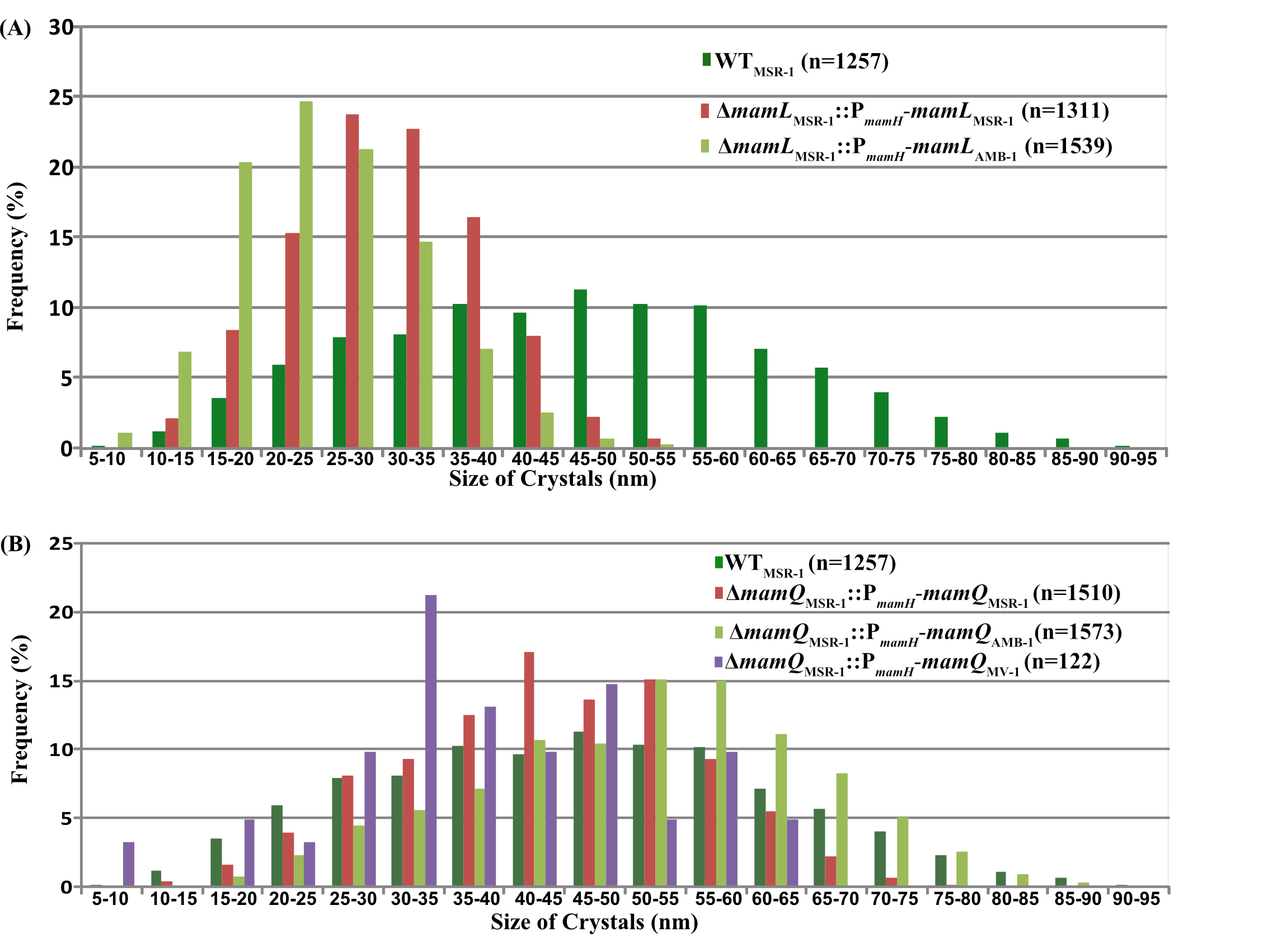

Supplement: Fig. S4 — Magnetosome distribution in the mutants with mamL and mamQ. [file mbio.03282-22-s0005.tif]

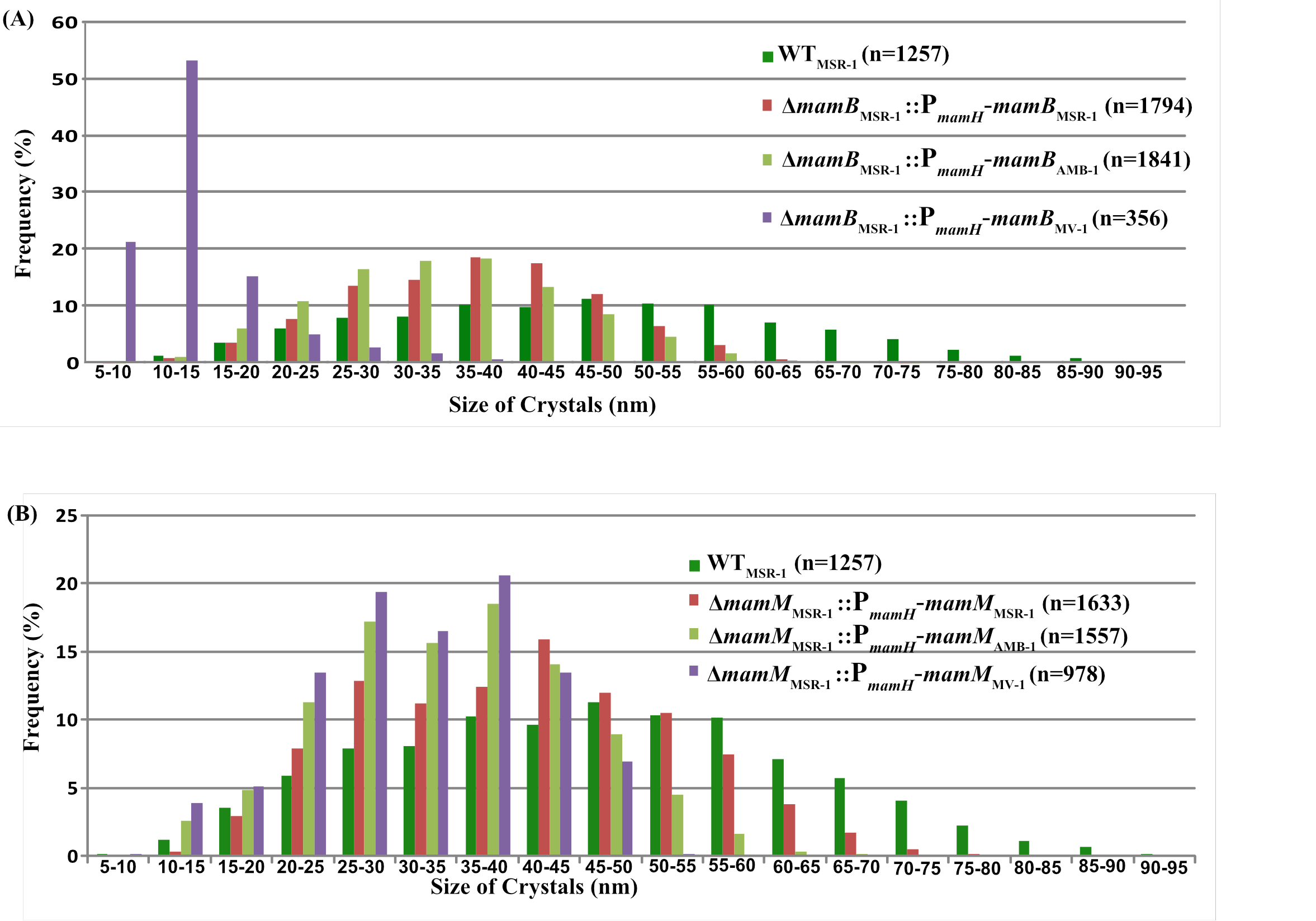

Supplement: Fig. S5 — Magnetosome size distribution in the mutants with mamB and ammM. [file mbio.03282-22-s0006.tif]

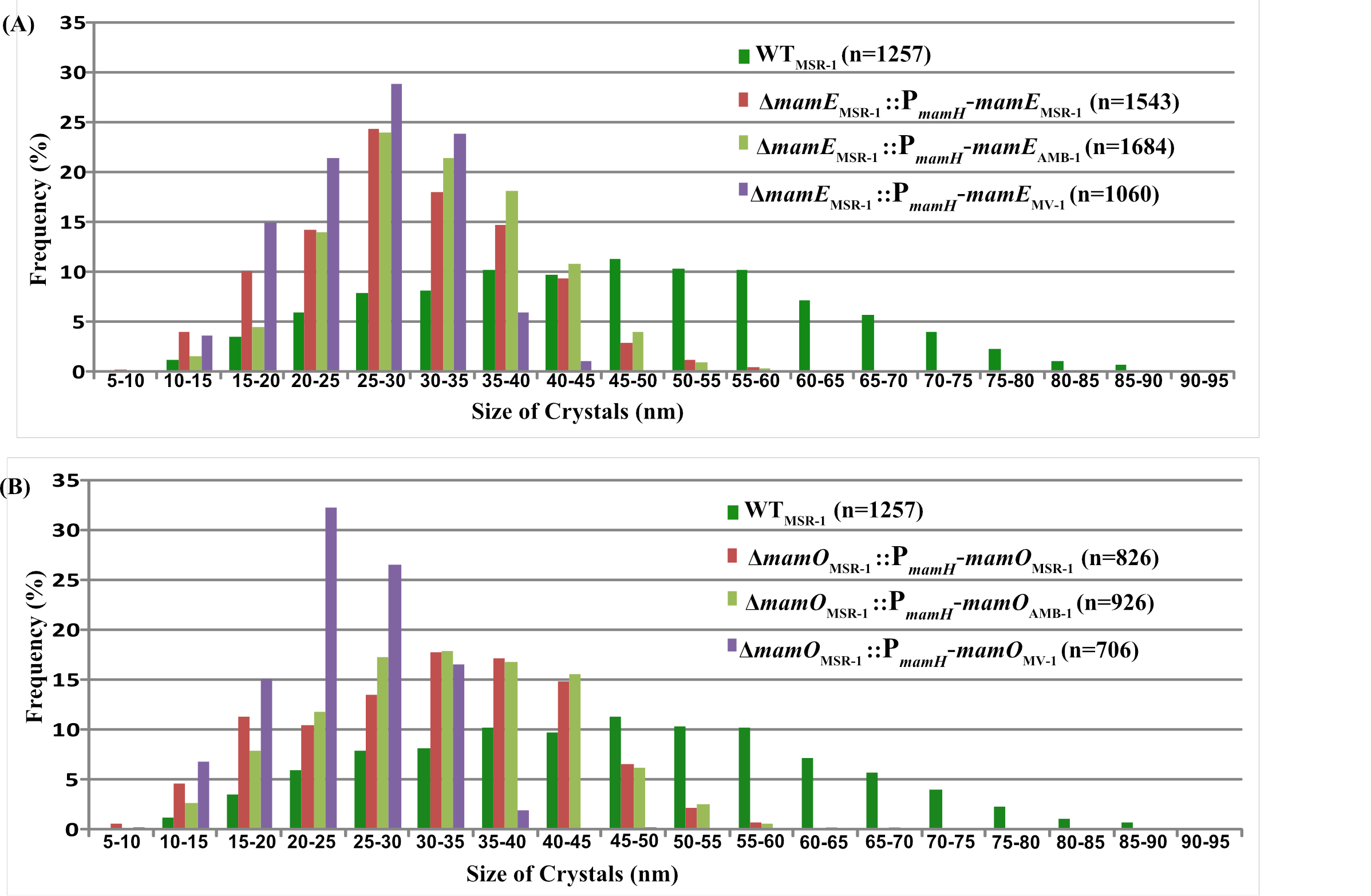

Supplement: Fig. S6 — Magnetosome size distribution in the mutants with mamE and mamO. [file mbio.03282-22-s0007.tif]

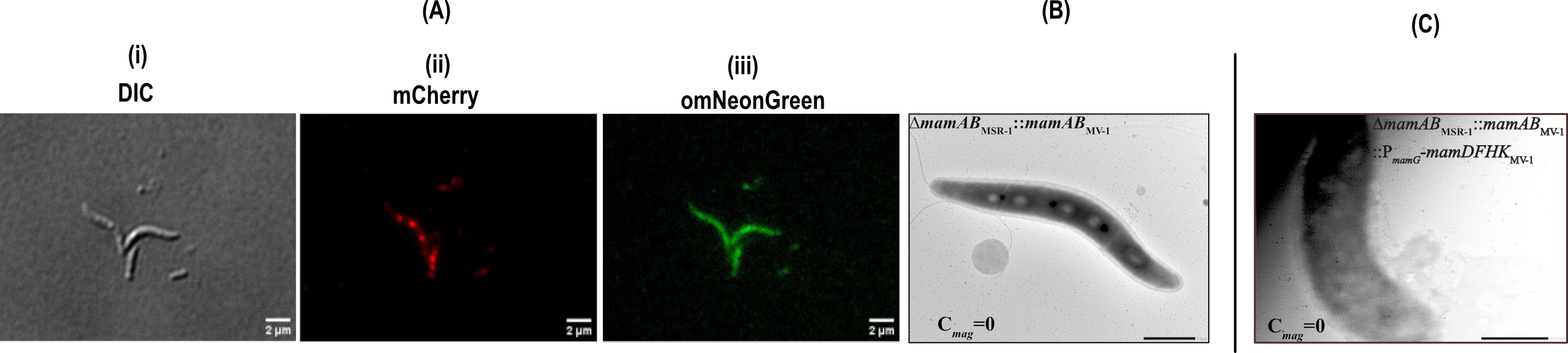

Supplement: Fig. S7 — Mutants with operons from MV-1. [file mbio.03282-22-s0008.tif]
