# Supplementary material for: Functional expression of foreign magnetosome genes in the alphaproteobacterium Magnetospirillum gryphiswaldense
Source: mBio. 2023 Jun 15;14(4):e03282-22. doi: 10.1128/mbio.03282-22 (PMC10470508; doi:10.1128/mbio.03282-22)
Supplement: Table S1 — Strains, primers, and plasmids used in this study. [file mbio.03282-22-s0009.docx]

**Table S1 A:** Strains used in this study.

| Bacterial strains | Characteristics | References |
| --- | --- | --- |
| *M. gryphiswaldense* (MSR-1) | Lab strain-Wildtype, *Rif^R^, Sm^R^* | (1) |
| ∆*mamQ*_MSR-1_ | deletion of MSR1_03450 | (2) |
| ∆*mamB*_MSR-1_ | deletion of MSR1_03470 | (3) |
| ∆*mamM*_MSR-1_ | deletion of MSR1_03400 | (4) |
| ∆A13_MSR-1_ | ∆*mms6*op, ∆*mamGFDC*op, ∆*mamXY*op | (5) |
| ∆*mamAB*_MSR-1_ | deletion of *mamAB*op in *MSR-1* | (5) |
| ∆MAI_AMB-1_ | deletion of MAI in AMB-1 | Dr. Marina Dziuba, manuscript in preparation |
| ∆*mamL*_MSR-1_ | deletion of MSR1_03390 | This study |
| ∆*mamE*_MSR-1_ | deletion of MSR1_03460 | This study |
| ∆*mamO*_MSR-1_ | deletion of MSR1_03420 | This study |
| ∆A13-∆*mamAB*op | deletion of *mamAB*op in ∆A13 | This study |
| *E. coli* Neb10ß | F- *mcrA* Δ(*mrr*-*hsd-*RMS*mcr*BC)  Φ80d*lac*ZΔM15  Δ*lac*X74 *end*A1 *rec*A1 *deo*R  Δ(*ara*,*leu*)7697 *ara*D139 *gal*  U *gal*K *nup*G *rps*L λ- | Purchased from NEB BioLabs Inc (MA, USA) |
| *E. coli* WM3064 | *thrB1004 pro thi rpsL hsdS lacZ∆M15 RP4-1360 ∆(araBAD)567 ∆dapA1341::[erm pir]* | William Metcalf, Universität von Illinois (UIUC), USA, unpublished |
| *S. cerevisiae* BY4741 | *MATa; his3Δ1; leu2Δ0; met15Δ0; ura3Δ0* | (6) |

**Table S1 B: Oligonucleotides used in this study. The sites recognized by restriction enzymes are underlined.**

| **Primer name** | **sequence 5'-3'** | **Purpose** | **Restriction Enzyme** |
| --- | --- | --- | --- |
|  | **Site-specific chromosomal deletion by homologous recombination** | |  |
| **RPA615** | GAGGTCGACATGAGTGAAGGTGAAGGCCAG | amplification of 5' flanking sequence of *mamL*_MSR-1_ | SalI |
| **RPA616** | TCAGCGCTTTCTTACCATACCAATGTCCTG |  | - |
| **RPA617** | ATGGTAAGAAAGCGCTGACATTCCATGCTCCG | amplification of 3' flanking sequence of *mamL* _MSR-1_ | - |
| **RPA618** | ATGGCGGCCGCCTAGTTATCCACCTTGGAC |  | NotI |
| **RPA603** | GAGGTCGACGTGACGGGAATGGAACCTG | amplification of 5' flanking sequence of *mamE* _MSR-1_ | SalI |
| **RPA604** | TCAAAGAACCATGGTCATCAACCATCGATGTTAGGGTCTGAG |  | - |
| **RPA605** | ATGACCATGGTTCTTTGAGGGTGGAGCGGGATAATGGC | amplification of 3' flanking sequence of *mamE*_MSR-1_ | - |
| **RPA606** | ATGGCGGCCGCCTATTTATTCTTATCTTCAGC |  | NotI |
| **RPA607** | ATGGGATCCGTGGTTGGATTTATCACCCTC | amplification of 5' flanking sequence of *mamO* _MSR-1_ | SalI |
| **RPA608** | TCACACCGTTTCAATCATGGCCGCTTTCC |  | - |
| **RPA609** | ATGATTGAAACGGTGTGATGGATATGGTTTTGGG | amplification of 3' flanking sequence of *mamO*_MSR-1_ | - |
| **RPA610** | ATAGCGGCCGCTTAGACGGCCGAACGTTCAT |  | NotI |
| **RPA611** | GTAGTCGACCTAATGCCGGACCAGGCTC | amplification of 5' flanking sequence of *mamAB_MSR-1_* | SalI |
| **RPA612** | TTATTTCGGAGGTTCCATTCCCGTCACAATTCACC |  | - |
| **RPA613** | GTGACGGGAATGGAACCTCCGAAATAACGCCGCCA | amplification of 3' flanking sequence of *mamAB* _MSR-1_ | - |
| **RPA614** | ATAGCGGCCGCCTACTGCGACAAGTTG |  | NotI |
| **RPA111** | CCCGACTTTTGATGGTTGCC | screening/sequencing primers for deletion constructs | - |
| **RPA112** | CGAACGCCTTCATCACCATG |  | - |
| **RPA113** | GGGCCACTGATAATGCTTGC |  | - |
| **RPA114** | CCAACACAATCCCAATGGCG |  | - |
| **RPA115** | CCCTCCTGCGCCTTTTATCT | screening/sequencing primers for *mamE* deletion construct and mutant | - |
| **RPA116** | ACCAGATCAGACACGGCAAG |  | - |
| **RPA117** | GTTCTGTTCTCGGCGCATTT |  | - |
| **RPA118** | CCGATATTCTCCACCGACCG |  | - |
| **RPA119** | GGCGTTACTCCCAATACCCC |  | - |
| **RPA120** | GACACGGCAAGCACAGAATC |  | - |
| **RPA121** | GCCATGAACCAATATTCCGGC | screening/sequencing primers for *mamO* deletion construct and mutant | - |
| **RPA122** | GGAACGAAATTGGGCAAGGG |  | - |
| **RPA123** | GCAAGGCGGTGTTGGAAATT |  | - |
| **RPA124** | GAACTTCTCGGTCGTCTGCA |  | - |
| **RPA105** | TGGTATCCGAGCCGTTCATG | screening/sequencing primers for *mamAB*op deletion construct and mutant | - |
| **RPA106** | CACCATCACGAACAATCCGC |  | - |
| **RPA107** | ATGATAGCCCCGACCGACTA |  | - |
| **RPA108** | AGTCAGCAACATGGTCACCG |  | - |
| **RPA109** | CTATCGGATTGTGGAGGCGG |  | - |
| **RPA110** | AGGGAAGGGAAGGGGGTATC |  | - |
|  | **Construction of pBAM-Tn5-based plasmids** | |  |
| **RPA298** | GGGAATTCCATATGGTGTCGAAGGGCGAGGAACTG | primers used for the construction of pBAM-Tn5-P*_mamDC_*_45_*_-_egfp*-HL for *N*-terminus fusion | Ndel |
| **RPA299** | CAGGCTAGCCTTATACAGCTCGTCCATGCC |  | - |
| **RPA300** | GCTGTATAAGGCTAGCCTGGCCGAAGC |  | - |
| **RPA301** | ATAGGATCCATAAGATATCGGCCGCGGCCTTCGC |  | BamHI/ EcoRV |
| **RPA302** | GGGAATTCCATATGACGCGTCGACCTGGCCGAAGCCGCGGCCAAG | primers used for the construction of pBAM-Tn5-P*_mamDC_*_45_-HL-*egfp* for *C*-terminus fusion | Ndel/SalI |
| **RPA303** | CGCGGATCCTCACTTATACAGCTCGTCCATGCCCAGGG |  | BamHI |
| **RPA304** | CTAGCTCTTAGTTCTCCAATAAATTCC | screening/sequencing primers for pBAM-Tn5 based plasmids | - |
| **RPA305** | CAAACTGCAATTTCATCTGATGCTG |  | - |
| **RPA682** | CCGCTCGAGGGCATCCTGATCGGTAGGCGATG | amplification of P*_mamH_* to construct pBAM-Tn5-PmamH-egfp-HL for *N*-terminus fusion | XhoI |
| **RPA683** | CCCAAGCTTTCCCGTCACAATTCACCTCCAATTC |  | HindIII |
|  | **Construction of pBAM-Tn5-P*_mamH_* based plasmids** | |  |
| **RPA1262** | GGGAATTCCATATGGTAAGAGTGATCGGATCG | amplification of *mamL* _MSR-1_ | Ndel |
| **RPA1263** | CGCGGATCCTCAGCGCTTAATGACGATG |  | BamHI |
| **RPA1264** | GGGAATTCCATATGGCAGTAAGCGATGCGGAC | amplification of *mamQ* _MSR-1_ | Ndel |
| **RPA1265** | CACCAGCGAGGTCATCATGTGCTTGTAGG |  | - |
| **RPA1266** | GCGGATACCTACAAGCACATGATGACCTC |  | - |
| **RPA1267** | GGCGGATCCTCAATTCTTGGATTCCTGC |  | BamHI |
| **RPA1268** | GGGAATTCCATATGAAGTTCGAAAATTGCAGAGACTGCCGGG | amplification of *mamB*_MSR-1_ | Ndel |
| **RPA1269** | GTCGGATCCTCAGACCCGGACCGTCACGGC |  | BamHI |
| **RPA1270** | GGGAATTCCATATGAGGAAGAGCGGTTGCGCGGTC | amplification of *mamM* _MSR-1_ | Ndel |
| **RPA1271** | CGCGGATCCCTAGTTATCCACCTTGGACAGCATG |  | BamHI |
| **RPA1272** | GGGAATTCCATATGACCATGTTCAATGGTGATGTGGAAGAC | amplification of *mamE* _MSR-1_ | Ndel |
| **RPA1273** | CGCGGATCCTCAAAGAACAATCCAGAACTCTTGG |  | BamHI |
| **RPA1274** | GGGAATTCCATATGATTGAAATTGGCGAGACCATG | amplification of *mamO* _MSR-1_ | Ndel |
| **RPA1275** | GACCACCAGCTAGAGGACCCCATGGCC |  | - |
| **RPA1276** | CCAAACTGTGGCCATGGGGTCCTCTAG |  | - |
| **RPA1277** | CGCGGATCCTCACACCGTTGTCAGCATCTTG |  | BamHI |
| **RPA1278** | TGATTGCTGCGGGTACTCC | sequencing primers for pBAM-Tn5-P*_mamH_*-*mamE* _MSR-1_ | - |
| **RPA1279** | CGAACTGGTGGCATGTGTTG |  | - |
| **RPA1280** | TATCAACATTGCCGCAACCG | sequencing primers for pBAM-Tn5-P*_mamH_*-*mamO* _MSR-1_ | - |
| **RPA1281** | AGAAGACCATCATGCCAGCG |  | - |
| **RPA1284** | GGGAATTCCATATGGTAAGATTGATCGGATCG | amplification of *mamL*_AMB-1_ | Ndel |
| **RPA1285** | CGCGGATCCTCAGCGCTTGATGACGATG |  | BamHI |
| **RPA1286** | GGGAATTCCATATGACGAAGGGGCCTCGGTCTG | amplification of *mamQ*_AMB-1_ | Ndel |
| **RPA1287** | CAGCGACGTCATCATGTGCTTGTAGGTG |  | - |
| **RPA1288** | GGTGGACACCTACAAGCACATGATGACG |  | - |
| **RPA1289** | GGAGGATCCTCATTTCTTGATGTCCTGCGCATG |  | BamHI |
| **RPA1290** | CCGAATTCCATATGAAGTTCGAAAATTGCAGGGATTGCCGCGAGGAAGTGG | amplification of *mamB*_AMB-1_ | Ndel |
| **RPA1291** | CTAGGATCCTCAGGCCCGTGCCGCGGCGGC |  | BamHI |
| **RPA1292** | GGGAATTCCATATGAGGAAGAGCGGTTGCACGG | amplification of *mamM*_AMB-1_ | Ndel |
| **RPA1293** | CGCGGATCCCTAGTTATCCACCTTCGACAAC |  | BamHI |
| **RPA1294** | GGGAATTCCATATGGCCATGTTCAATGGTGACG | amplification of *mamE*_AMB-1_ | Ndel |
| **RPA1295** | CGCGGATCCTCAAAGGACAATCCAGAACTC |  | BamHI |
| **RPA1296** | GGGAATTCCATATGATTGAAGTCGGCGAGACCATG | amplification of *mamO*_AMB-1_ | Ndel |
| **RPA1297** | GAATCAGACCCGTGCTGGAACCGTGGAG |  | - |
| **RPA1298** | GGCCTTCCTCCACGGTTCCAGCACGGGTC |  | - |
| **RPA1299** | CGCGGATCCTCACACCGAGGTCAGCATCTTG |  | BamHI |
| **RPA1300** | TCTTGGATGAAGTGGGCTGG | sequencing primers for pBAM-Tn5-P*_mamH_*-*mamE*_AMB-1_ | - |
| **RPA1301** | GGTGCAATCCATGTTCTGGC |  | - |
| **RPA1302** | CAAGGCAATGTGGTCAAGGG | sequencing primers for pBAM-Tn5-P*_mamH_*-*mamO*_AMB-1_ | - |
| **RPA1303** | TGACCAGGCCGACGATATC |  | - |
| **RPA1238** | CCCAATTCCATATGCTTCTCTATTTGGTGATC | amplification of *mamL*_MV-1_ | Ndel |
| **RPA1239** | GGAGGATCCTTATTCAGGGCGTGATTGC |  | BamHI |
| **RPA1240** | GGGAATTCCATATGCGAGGCGTGAAGGTGATG | amplification of *mamQ*_MV-1_ | Ndel |
| **RPA1241** | CGCGGATCCTCATTGATTTCCCCCATTTTCTTCCAGG |  | BamHI |
| **RPA1242** | GGGAATTCCATATGAAATCAGAACGGTGCAAAATTTG | amplification of *mamB*_MV-1_ | Ndel |
| **RPA1243** | GATGGATCCTCAGCTAAACCAGGTGGGCAACAAC |  | BamHI |
| **RPA1244** | GGGAATTCCATATGAGATACGAAAAGTGCATTAAG | amplification of *mamM*_MV-1_ | Ndel |
| **RPA1245** | TGCAACGATGGGTCCATCAGG |  | - |
| **RPA1246** | CCTGATGGACCCATCGTTGCACCGC |  | - |
| **RPA1247** | CGCGGATCCTTAGTCCTTCGTCTCAGTTTCTG |  | BamHI |
| **RPA1248** | GGGAATTCCATATGAGCGACAACAGTGAGTGGG | amplification of *mamE*_MV-1_ | Ndel |
| **RPA1249** | CGCGGATCCTTAGGGCAACACCACAAAAAACTC |  | BamHI |
| **RPA1250** | CCCAATTCCATATGAAGACTATAGAACAACCCAC | amplification of *mamO*_MV-1_ | Ndel |
| **RPA1251** | CGCGGATCCTTAGTTCAAAAACAACATTTTAACG |  | BamHI |
| **RPA1252** | TCAAAACACCTACGCCGTTG | sequencing primer for pBAM-Tn5-P*_mamH_*-*mamE*_MV-1_ | - |
| **RPA1253** | CACAAATTCGCCGGGCTTC | sequencing primer for pBAM-Tn5-P*_mamH_*-*mamO*_MV-1_ | - |
| **RPA1226** | GGGAATTCCATATGTTCAGGTTGCTGCTATCACTG | amplification of *mamL*_MC-1_ | Ndel |
| **RPA1227** | CGCGGATCCTCAGAACTCATCCACATCTTTTTTAC |  | BamHI |
| **RPA1228** | GGGAATTCCATATGAGTAAAAATGGTCGCGACGC | amplification of *mamQ*_MC-1_ | Ndel |
| **RPA1229** | GGGGGATCCTCATGGCGCTCTCTTGTCATTGGAC |  | BamHI |
| **RPA1230** | GGATGGTAGTGTGGTCGGTG | sequencing primer for pBAM-Tn5-P*_mamH_*-*mamE*_MC-1_ | - |
| **RPA1231** | CTAGGGCTGGTTTGGGTGG | sequencing primer for pBAM-Tn5-P*_mamH_*-*mamO*_MC-1_ | - |
| **RPA1176** | GGGAATTCCATATGTTAAGAACGATAGTGAC | amplification of *mamL_RS_*_-1_ | Ndel |
| **RPA1177** | CGCGGATCCTTAATCCAAGATATCGTCG |  | BamHI |
| **RPA1178** | GGGAATTCCATATGGCCGAGATGCCCATGGATG | amplification of *mamQ*_RS-1_ | Ndel |
| **RPA1179** | CGCGGATCCCTAGTAACTGATGGGCGTCAGCACC |  | BamHI |
| **RPA1180** | GGGAATTCCATATGAAATACAAGGCATGTGAAAAGTGCGC | amplification of *mamB_RS_*_-1_ | Ndel |
| **RPA1181** | CGCGGATCCTCATCCTTCGGCGGGGTTAAGAAC |  | BamHI |
| **RPA1182** | GGGAATTCCATATGCGAAAAGTCTGTCAGG | amplification of *mamM_RS_*_-1_ | Ndel |
| **RPA1183** | GATGGATCCTCATGCTTGCGCCCCACAC |  | BamHI |
| **RPA1200** | CCCAATTCCATATGTCAGATACTTTTGAAGAATTAAACGCACCTGGTTG | amplification of *mamEO_RS_*_-1_ | Ndel |
| **RPA1201** | GATGGATCCCTAGCGCGCCTGTCCGGACAGGAG |  | BamHI |
| **RPA1192** | GGCACCGAGACGAACAAATG | sequencing primer for pBAM-Tn5-P*_mamH_*-*mamEO*_RS-1_ | - |
| **RPA1193** | TCCAGACTGGCGATGTGATC |  | - |
| **RPA1194** | ATGCTTGGCCCGATTGATGG |  | - |
| **RPA1195** | CGACATGATCCAGACCGACG |  | - |
| **RPA1196** | GGTAGCCAAACAAACCGACC |  | - |
| **RPA1197** | TCTCGACCCGACCTATCTGC |  | - |
|  | **Construction of pBAM-Tn5-P*_mamDC45_*-egfp (N/C termini) based plasmids** | | |
| **RPA338** | ATGGTAAGAGTGATCGGATCGTTG | amplification of *mamL* _MSR-1_ | - |
| **RPA339** | ATCTCAGCGCTTAATGACGATGTTTTTCC |  | - |
| **RPA2171** | ATGGCAGTAAGCGATGCGGAC | amplification of *mamQ* _MSR-1_ | - |
| **RPA2172** | CATGGATCCTCAATTCTTGGATTCCTGCGAATG |  | BamHI |
| **RPA2117** | CCCTGCGAAGCTTAGGAGATCAGCATATGAAGTTCGAAAATTGCAGAGACTGCCGGG | amplification of *mamB* _MSR-1_ | - |
| **RPA2118** | GCGGCTTCGGCCAGGTCGACGACCCGGACCGTCACGGC |  | - |
| **RPA2119** | CCTGCGAAGCTTAGGAGATCAGCATATGAGGAAGAGCGGTTGC | amplification of *mamM* _MSR-1_ | - |
| **RPA2120** | GCGGCTTCGGCCAGGTCGACGTTATCCACCTTGGACAGCATG |  | - |
| **RPA2173** | ATGACCATGTTCAATGGTGATGTG | amplification of *mamE* _MSR-1_ | - |
| **RPA2174** | CAGGGATCCTCAAAGAACAATCCAGAACTCTTG |  | BamHI |
| **RPA2175** | ATGATTGAAATTGGCGAGACCATGGGTGATC | amplification of *mamO* _MSR-1_ | - |
| **RPA2176** | CTAGAGGACCCCATGGCCACAG |  | - |
| **RPA2177** | CATGGGGTCCTCTAGCTGGTG |  | - |
| **RPA2178** | CGCGGATCCTCACACCGTTGTCAGCATCTTGATGG |  | BamHI |
| **RPA314** | ATGCTTCTCTATTTGGTGATCGTC | amplification of *mamL*_MV-1_ | - |
| **RPA315** | ATCTTATTCAGGGCGTGATTGCAG |  | - |
| **RPA1518** | ATGGTTACTATTATAGATCAATCG | amplification of *mamQ*_MV-1_ | - |
| **RPA1519** | ATCTCATTGATTTCCCCCATTTTC |  | - |
| **RPA1514** | GGGAATTCCATATGAAATCAGAACGGTGC | amplification of *mamB*_MV-1_ | Ndel |
| **RPA1515** | CTATGTCGACGCTAAACCAGGTGGGCAAC |  | SalI |
| **RPA1516** | GGGAATTCCATATGAGATACGAAAAGTGCATTAAG | amplification of *mamM*_MV-1_ | Ndel |
| **RPA1517** | CGACGTCGACGTCCTTCGTCTCAGTTTCTG |  | SalI |
| **RPA688** | ATGAGCGACAACAGTGAGTGGGGC | amplification of *mamE*_MV-1_ | - |
| **RPA2188** | CGCGGATCCTTAGTTCAAAAACAACATTTTAACGGCGACTGC |  | BamHI |
| **RPA696** | ATGAAGACTATAGAACAACCCACAAG | amplification of *mamO*_MV-1_ | - |
| **RPA2189** | CGCGGATCCTTAGTTCAAAAACAACATTTTAACGGCGAC |  | BamHI |
| **RPA1504** | ATGTTCAGGTTGCTGCTATCAC | amplification of *mamL*_MC-1_ | - |
| **RPA1505** | ATCTCAGAACTCATCCACATC |  | - |
| **RPA1506** | ATGAGTAAAAATGGTCGCGACG | amplification of *mamQ*_MC-1_ | - |
| **RPA1507** | ATCTCATGGCGCTCTCTTGTC |  | - |
| **RPA1500** | GGGAATTCCATATGAAGTACGATGAATGTCGAAATTGC | amplification of *mamB*_MC-1_ | Ndel |
| **RPA1501** | CTAAGTCGACACGGGACTCCGTACGCAG |  | SalI |
| **RPA1502** | GGGAATTCCATATGCGTTACTCTAAATGCATCGTC | amplification of *mamM*_MC-1_ | Ndel |
| **RPA1503** | CGACGTCGACACTCTTGGCCAGATCTGTTAATAC |  | SalI |
| **RPA1508** | ATGACACCCGAGCAAGATCCCAG | amplification of *mamE*_MC-1_ | - |
| **RPA1509** | ATCTCACTGTACTTTAACGAACATCCG |  | - |
| **RPA1510** | ATGGCATTCTCCCCCATGAAG | amplification of *mamO*_MC-1_ | - |
| **RPA1511** | ATCTCATACAACGAACATCCG |  | - |
| **RPA1524** | ATGTTAAGAACGATAGTGACAGTGGTCCTGAC | amplification of *mamL_RS_*_-1_ | - |
| **RPA1525** | ATCTTAATCCAAGATATCGTCGACGTC |  | - |
| **RPA1526** | ATGGCCGAGATGCCCATGGATGTC | amplification of *mamQ_RS_*_-1_ | - |
| **RPA1527** | ATCCTAGTAACTGATGGGCGTCAGC |  | - |
| **RPA1180** | GGGAATTCCATATGAAATACAAGGCATGTGAAAAGTGCGC | amplification of *mamB_RS_*_-1_ | Ndel |
| **RPA1522** | CGACGTCGACTCCTTCGGCGGGGTTAAGAAC |  | SalI |
| **RPA1182** | GGGAATTCCATATGCGAAAAGTCTGTCAGG | amplification of *mamM*_RS-1_ | Ndel |
| **RPA1664** | GCCGGGTCCACGAAGATGTCTACG |  | - |
| **RPA1665** | CATCTTCGTGGACCCGGCCAACACAG |  | - |
| **RPA1523** | CTAAGTCGACTGCTTGCGCCCCACACATC |  | SalI |
| **RPA1528** | ATGGCAGCATGGCGGATTGTCTGGAC | amplification of *mamEO_RS_*_-1_ | - |
| **RPA2190** | CATGGATCCTCATATCAGATTGGCTATCCGCAAGATC |  | BamHI |
|  | **Construction of pTps-based plasmid** | |  |
| **RPA266** | GCGTCACACCGTGTACCGTTCGTATAGCATAC | primers used to construct pTps-Kn^R^-RPA | DraIII |
| **RPA267** | GCATAAGCTTTTAATTAAGGCCGCCTAGGCCGC |  | HindIII |
| **RPA268** | CAGCAAGCGAACCGGAATTG | sequencing primers for pTps-Kn^R^-RPA | - |
| **RPA968** | CGAGCCTGCAAAAACGTCTG |  | - |
| **RPA969** | GCCGTTACTAGACCGTCCAG |  | - |
| **RPA970** | GTTCCACCAAGACAACGCAC |  | - |
| **RPA971** | ACACGTGCTGATCAGATCCG |  | - |
| **RPA972** | CGTTTTTCCATAGGCTCCGC |  | - |
| **RPA670** | GGTTGGGAAGCCCTGCAACGTATAATATTTGCCCATGGACGCACACCGTGCCAGCTGTCTCTTATACACATCTGACGTC | primers for exchange of Kn^R^ with Cm^R^ in pTps-Kn^R^-RPA resulting pTps-Cm^R^-RPA | - |
| **RPA671** | GCCGCGCGCGGAGACGAGACGAGACAGCCTGAGAATGGATGCGAGTAATGAAGTTTAAACTTTACCGTTCGTATAATGTATGCTATACG |  | - |
| **RPA704** | AGCATACATTATACGAACGGTAGTTTCATTACTCGCATCCATTCTCAGGC | primers for construction of pTps-Kn_R_-P*_mamG_*-*mamB*_MV-1_-P*_mamH_*-*mamM*_MV-1_ | - |
| **RPA705** | CGACAAATTTTGCACCGTTCTGATTTCATATGACCCTCCGGCAAGTGTATG |  | - |
| **RPA706** | GGAATCGTGCATACACTTGCCGGAGGGTCATATGAAATCAGAACGGTGCAAAATTTGTC |  | - |
| **RPA707** | CTTACTATTGCTGGCAGGAGGTCAGGCCCGGGCTCAGCTAAACCAGGTGGGCAAC |  | - |
| **RPA708** | CAAGTTGTTGCCCACCTGGTTTAGCTGAGCCCGGGCCTGACCTCCTGC |  | - |
| **RPA709** | CCTGGAGCACTTAATGCACTTTTCGTATCTCATTCCCGTCACAATTCACCTCC |  | - |
| **RPA710** | GCGAATTGGAGGTGAATTGTGACGGGAATGAGATACGAAAAGTGCATTAAG |  | - |
| **RPA711** | GTTGTGGTAATCTATGTATCCTGGCGCGCGCGCTTAGTCCTTCGTCTCAGTTTCTGG |  | - |
| **RPA1676** | CATTACTCGCATCCATTCTCAGGCTGTCTCGTCTCGTCTCCGCGCGCGGCCAACTTTTTCGCTTTACTAGCTCTTAG | primers for construction of pTps-Kn_R_-P*_mamG_*-*mamB*_MC-1_-P*_mamH_*-*mamM*_MC-1_ | - |
| **RPA1677** | CGGCAATTTCGACATTCATCGTACTTCATCTGATCTCCGGCAAGTGTATGCACGATTCCC |  | - |
| **RPA1678** | CGTGCATACACTTGCCGGAGATCAGATGAAGTACGATGAATGTCGAAATTGCCGGGATAC |  | - |
| **RPA1679** | CAAATAAAAACCCACCAATCTGAACTCAACGGGACTCCGTACGCAGCGGTGTCAAAAATATC |  | - |
| **RPA1680** | CACCGCTGCGTACGGAGTCCCGTTGAGTTCAGATTGGTGGGTTTTTATTTGATTACATTGG |  | - |
| **RPA1681** | CAGACGATGCATTTAGAGTAACGCATAAACGTTCTCCAGTCTTGATACAGAACAC |  | - |
| **RPA1682** | CTGTATCAAGACTGGAGAACGTTTATGCGTTACTCTAAATGCATCGTCTGCTATGAAATGATTGGTTGGGCC |  | - |
| **RPA1683** | GAAGGGCTCGGAGTTGTGGTAATCTATGTATCCTGGCGCGCGCGCTCAACTCTTGGCCAGATCTGTTAATAC |  | - |
| **RPA1676** | CATTACTCGCATCCATTCTCAGGCTGTCTCGTCTCGTCTCCGCGCGCGGCCAACTTTTTCGCTTTACTAGCTCTTAG | primers for construction of pTps-Kn_R_-P*_mamG_*-*mamB*_RS-1_-P*_mamH_*-*mamM*_RS-1_ | - |
| **RPA1684** | CGCACTTTTCACATGCCTTGTATTTCATCTGATCTCCGGCAAGTGTATGCACGATTCCCTCTC |  | - |
| **RPA1685** | CGTGCATACACTTGCCGGAGATCAGATGAAATACAAGGCATGTGAAAAGTGCGCCCGG |  | - |
| **RPA1686** | GTAATCAAATAAAAACCCACCAATCTGAACTCATCCTTCGGCGGGGTTAAGAACGACTGTAATCC |  | - |
| **RPA1687** | GTCGTTCTTAACCCCGCCGAAGGATGAGTTCAGATTGGTGGGTTTTTATTTGATTACATTGGCC |  | - |
| **RPA1688** | GGCAATCCTGACAGACTTTTCGCATAAACGTTCTCCAGTCTTGATACAGAACACAAGCGGCG |  | - |
| **RPA1689** | GCTTGTGTTCTGTATCAAGACTGGAGAACGTTTATGCGAAAAGTCTGTCAGGATTGCCTCAAATGCGTC |  | - |
| **RPA1690** | CTCGGAGTTGTGGTAATCTATGTATCCTGGCGCGCGCGCTCATGCTTGCGCCCCACACATCGTAC |  | - |
| **RPA449** | CCGGATATGAACAAACTGCAATTTC | sequencing primers for co-expression constructs | - |
| **RPA627** | CATTACTCGCATCCATTCTCAGG |  | - |
| **RPA712** | GTGACGTCTTGTTTCGAGCAGATGC |  | - |
| **RPA713** | CACCTCCAATTCGCACGGACTATAG |  | - |
| **RPA638** | GTGGAAGGGCTCGGAGTTGTG |  | - |
| **RPA270** | CATTACTCGCATCCATTCTCAGGCTGTCTCGTCTCGTCTCTGTTTATGTGTTGTCAACCGCCCCCGAC | primers for construction of pTps-Kn^R^-*mamAB*_AMB-1_ | - |
| **RPA1125** | CGTCGCCTTTGTCGCGATCTCGCCGGTTATTCGCCATTATCCAGCTCCACCATCAAAGGACAATC |  | - |
| **RPA1126** | GTCCTTTGATGGTGGAGCTGGATAATGGCGAATAACCGGCGAGATCGCGACAAAGGCGACGGGAGCC |  | - |
| **RPA1127** | CCTCATCCTTACTCACTCCAAAGCCCCGACCCCGCCTTCAACCCAAGCGAAGGGATTCAGCGC |  | - |
| **RPA1128** | GAATCCCTTCGCTTGGGTTGAAGGCGGGGTCGGGGCTTTGGAGTGAGTAAGGATGAGGAAGAGC |  | - |
| **RPA1129** | CTGAGTTCCAAGCTCATTCGGATTCCTGTCGTCATCTCTCATCCCACGAGAACC |  | - |
| **RPA1130** | GATGAGAGATGACGACAGGAATCCGAATGAGCTTGGAACTCAGCCATGATTG |  | - |
| **RPA1131** | CGCCTCTCTAAGCTCGTCGTATGAAGGAAACGCCCCACATACGGCCTCAGAC |  | - |
| **RPA1132** | GGCCGTATGTGGGGCGTTTCCTTCATACGACGAGCTTAGAGAGGCGGATATGG |  | - |
| **RPA1133** | CCGCCCCGTCCCGTCCCTGGTCAGCGGGGAATGGCGCCCGCGATCATAATTG |  | - |
| **RPA1134** | CAATTATGATCGCGGGCGCCATTCCCCGCTGACCAGGGACGGGACGGGGCGGAGC |  | - |
| **RPA281** | GGTGGAAGGGCTCGGAGTTGTGGTAATCTATGTATCCTGGCGAGGCAGGGTCCTTCTTCAAAAAGAAATACGCC |  | - |
| **RPA216** | CATTACTCGCATCCATTCTCAGGCTGTCTCGTCTCGTCTCCGCGCGCGGCTGAAAACTTGCAACAAACTCGGCATCC | primers for construction of pTps-Kn^R^-*mamAB*_MV-1_ | - |
| **RPA207** | TATGTTTATCAGCCGATCTGAATAAAATTGGGTTTGAAG |  | - |
| **RPA208** | GATCGGCTGATAAACATATTACATAATCCAGTGAAAC |  | - |
| **RPA209** | AAACACGGCAATAGAATGAGACGTCTCAGGGCG |  | - |
| **RPA210** | TCTCATTCTATTGCCGTGTTTTAAAGTTCGGCCTGTTCG |  | - |
| **RPA211** | ACGTTAAGGACAAGTGGTGATGAAGACTATAGAACAAC |  | - |
| **RPA212** | CACCACTTGTCCTTAACGTGCCACGATCATAATG |  | - |
| **RPA213** | GTAAATAAGATGGACATGGATATGGACAGCACC |  | - |
| **RPA214** | TCCATGTCCATCTTATTTACCCGTCTTTGC |  | - |
| **RPA217** | GGTGGAAGGGCTCGGAGTTGTGGTAATCTATGTATCCTGGCGCGCGCGCAATTGCCTACTCCTGTAGAGATGAACCATCCGAGG |  | - |
| **RPA238** | GATAATCCGGATATGAACAAACTGC | screening and sequencing primers for pTps-Kn^R^-*mamAB*op_MV-1_ | - |
| **RPA239** | GTTGCAAGCCCAGGAAGAGAAAA |  | - |
| **RPA240** | CGACCTTTTCAAGCTGAGCCA |  | - |
| **RPA241** | TGCTTGATGTCGAAGGGGTC |  | - |
| **RPA242** | GCTTGACGATGACTTTGGCC |  | - |
| **RPA243** | TGACGTGGTGGTGAAAGTCC |  | - |
| **RPA244** | ACTGTTCATCTCGTTGCCCC |  | - |
| **RPA245** | CGCCATTGAAACCCCCAAAG |  | - |
| **RPA246** | TACCACCGGCAACAATCCAATTTTC |  | - |
| **RPA247** | TGGAGTCTACTGGCCGATTG |  | - |
| **RPA248** | TTTCTTCCAGGAACGGCACC |  | - |
| **RPA249** | CGCACACCATGGTTCTTTCG |  | - |
| **RPA250** | TACGGCCTGCTGATACTTGC |  | - |
| **RPA251** | CTCTGTCCCCATGCAACGTA |  | - |
| **RPA252** | ACGCCCAGAAGACAATCACG |  | - |
| **RPA253** | TCACCCCGGATCGCTTTATG |  | - |
| **RPA254** | GAGATTTGCAGCGTCCCAAC |  | - |
| **RPA255** | TCGATTTCCTCTCCGGCATG |  | - |
| **RPA256** | TCTACCTCGGCGCTTTTCAG |  | - |
| **RPA257** | GTTGCAGGGTCACAGGCTAT |  | - |
| **RPA258** | CCACGTCTTTGGCCGAATAC |  | - |
| **RPA259** | GCGTCATTTGGTCAGCCATG |  | - |
| **RPA260** | GCAAACTTCATCGTCGGGAC |  | - |
| **RPA261** | GTTTGAAGGCGGGCGATATC |  | - |
| **RPA262** | CCAGCCGTCATGTCCTTGAT |  | - |
| **RPA263** | GGTCCGTCGATTCTCAAGGG |  | - |
| **RPA264** | CCACCATCTGCGCATCCAC |  | - |
| **RPA265** | ACTGGGTGTAGCGTCGTAAG |  | - |
| **RPA2090** | CAGGCTGTCTCGTCTCGTCTCCGCGCGCGGCGTTCAGATTGGTGGGTTTTTATTTGATTACATTGG | primers used for construction of pTps-Kn^R^-P_MSR-1_-*mamAB*op_MV-1_-RG | - |
| **RPA2091** | CCAAAAATGACGACGTTCATATGCTGATCTCCTAAGCTTAAACGTTCTCCAGTCTTGATACAGAACAC |  | - |
| **RPA2092** | GTATCAAGACTGGAGAACGTTTAAGCTTAGGAGATCAGCATATGAACGTCGTCATTTTTGGATTGC |  | - |
| **RPA2093** | GTTATCCTCCTCGCCCTTGCTCACCATTTCAGGGCGTGATTGCAGAAATTTGCTTTCATC |  | - |
| **RPA2094** | CAAATTTCTGCAATCACGCCCTGAAATGGTGAGCAAGGGCGAGGAGGATAACATGGC |  | - |
| **RPA2095** | GTCCTGAATGTTTAAATGAGGCTGCCTTCCGTTTACTTGTACAGCTCGTCCATGCCGCCGG |  | - |
| **RPA2096** | CGGCATGGACGAGCTGTACAAGTAAACGGAAGGCAGCCTCATTTAAACATTCAGGACGCGC |  | - |
| **RPA2203** | GCACTTAATGCACTTTTCGTATCTCATAAGGGCTGCTCCCGTGGTGGCTGTGGTGGTCGC |  | - |
| **RPA2204** | GACCACCACAGCCACCACGGGAGCAGCCCTTATGAGATACGAAAAGTGCATTAAGTGCTCCAG |  | - |
| **RPA2205** | GTAAACAGCTCCTCGCCCTTGCTCATAAGTTCGGCCTGTTCGTTAGCGCGTTTAAAGTATGC |  | - |
| **RPA2206** | CTTTAAACGCGCTAACGAACAGGCCGAACTTATGAGCAAGGGCGAGGAGCTGTTTACCG |  | - |
| **RPA2101** | GACGTAAATCACCCGAGGCCGAACCAGTGCGTTATTTATATAGCTCGTCCATACCCAAGGTG |  | - |
| **RPA2102** | CCTTGGGTATGGACGAGCTATATAAATAACGCACTGGTTCGGCCTCGGGTGATTTACGTCTGATGCC |  | - |
| **RPA2103** | CGCCATCACCTTCACGCCTCGCATTGGCTCCGCTTCCGCTAGCTGCGCGGCCTG |  | - |
| **RPA2104** | GCGCAGCTAGCGGAAGCGGAGCCAATGCGAGGCGTGAAGGTGATGGCGGTGATTTC |  | - |
| **RPA2105** | GTCTTCCTCGCCCTTGGACACCATGCTAAACCAGGTGGGCAACAACTTGCTC |  | - |
| **RPA2106** | CAAGTTGTTGCCCACCTGGTTTAGCATGGTGTCCAAGGGCGAGGAAGACAATATGG |  | - |
| **RPA2107** | GTATACCTCGTTCAATTCACGCAAATCACTTGTACAGTTCGTCCATGCCCATC |  | - |
| **RPA2108** | GGCATGGACGAACTGTACAAGTGATTTGCGTGAATTGAACGAGGTATACGCACG |  | - |
| **RPA2109** | GTAATCTATGTATCCTGGCGCGCGCGCCTACTCCTGTAGAGATGAACCATCCGAGGTC |  | - |
| **RPA2110** | CGGATGGTTCATCTCTACAGGAGTAGGCGCGCGCGCCAGGATACATAGATTACCACAACTC |  | - |
| **RPA2111** | GTAATCAAATAAAAACCCACCAATCTGAACGCCGCGCGCGGAGACGAGACGAGACAGCCTGAGAATGG |  | - |
| **RPA623** | CATTCTCAGGCTGTCTCGTCTCGTCTCCGCGCGCGGCTGAAGCCACCTTGACAGAAATTGATATC | primers used to construct pTps-Cm^R^-P*_mamG-_mamDFHK*op_MV-1_ | - |
| **RPA624** | GCTTTGGTCATTTTCAAAAGATACATATGACCCTCCGGCAAGTGTATGCACGATTCCCTCTC |  | - |
| **RPA625** | GTGCATACACTTGCCGGAGGGTCATATGTATCTTTTGAAAATGACCAAAGCTTCG |  | - |
| **RPA626** | GGAGTTGTGGTAATCTATGTATCCTGGCGCGCGCGCCTAATTATCAAGAAAATCTAGAGTCGATTTC |  | - |
| **RPA627** | CATTACTCGCATCCATTCTCAGG | sequencing primers for pTps-Cm^R^-P*_mamG_*-*mamDFHK*op_MV-1_ | - |
| **RPA628** | GCCACGGGTTTTGATTTGGTTG |  | - |
| **RPA629** | CCCATCCCCCTCGCCATC |  | - |
| **RPA630** | CACCCGTCGTAGCACCCC |  | - |
| **RPA631** | AGAGAATGAAGCTGGCCGC |  | - |
| **RPA632** | CGCCGACACCCAAAAGAATC |  | - |
| **RPA633** | CAACGCCAACATCCAAGTGG |  | - |
| **RPA634** | GCGCCGAGGTGTTTTATCG |  | - |
| **RPA635** | GTCAGACATGGCAGAGTGGG |  | - |
| **RPA636** | ACCTGAACTTCGTCCACCAC |  | - |
| **RPA637** | GTCGCCCGTCATTTGGTCAG |  | - |
| **RPA638** | GTGGAAGGGCTCGGAGTTGTG |  | - |
|  | **construction of pTps-TAR_RPA plasmid** | |  |
| **RPA1055** | GCTCAAGATGCCCCTGTTCTCATTTCCGATCGAAATGGGTAATAACTGATATAATTAAATTGAAGCTC | primers used to create pTps-TAR-RPA | - |
| **RPA1056** | CGGCAGCTGGCAACCTGACTTGTATCGTCGAAAAGTGCCACCTGGGTCCTTTTCATCAC |  | - |
| **RPA1002** | TTACCAATGTTTAATCAGGCTCGCG | sequencing primers for pTps-TAR-RPA | - |
| **RPA1003** | CGAAAAAGTGCCACCTGGGTC |  | - |
| **RPA1004** | TGCAGATCGAGAAGCACCTG |  | - |
| **RPA1005** | AAAAACGGAATCAGCGCCAC |  | - |
| **RPA1006** | ACAAGGGAGACGCATTGGG |  | - |
| **RPA1007** | ACCTTGTGCAGAACTCGTGG |  | - |
|  | **Construction of pTps-TAR-RPA based plasmids** | |  |
| **RPA892** | CATCCATTCTCAGGCTGTCTCGTCTCGTCTCCGCGCGCGGCCCCATCCGCCGTGGGGTGTTTTCGGCCCCGC | primers to construct PTps-MAG_AMB-1_ native constructs-TAR | - |
| **RPA877** | GCTATCCATCAGGCTCCGCTCCCACTGGCTTCGCTGCCGCCGATCAGGC |  | - |
| **RPA878** | GGCAGCGAAGCCAGTGGGAGCGGAGCCTGATGGATAGCAACGAAAAAGAACAACCC |  | - |
| **RPA879** | GTCTCGATTTCCTGCCGCCACCCTCGGATTCGGATCAGATGCTCTCTTCACCAGAAGATAAC |  | - |
| **RPA1124** | GAGCATCTGATCCGAATCCGAGGGTGGCGGCAGGAAATCGAGACTTTTTGTTTCGAGC |  | - |
| **RPA1125** | CGTCGCCTTTGTCGCGATCTCGCCGGTTATTCGCCATTATCCAGCTCCACCATCAAAGGACAATC |  | - |
| **RPA1126** | GTCCTTTGATGGTGGAGCTGGATAATGGCGAATAACCGGCGAGATCGCGACAAAGGCGACGGGAGCC |  | - |
| **RPA1127** | CCTCATCCTTACTCACTCCAAAGCCCCGACCCCGCCTTCAACCCAAGCGAAGGGATTCAGCGC |  | - |
| **RPA1128** | GAATCCCTTCGCTTGGGTTGAAGGCGGGGTCGGGGCTTTGGAGTGAGTAAGGATGAGGAAGAGC |  | - |
| **RPA1129** | CTGAGTTCCAAGCTCATTCGGATTCCTGTCGTCATCTCTCATCCCACGAGAACC |  | - |
| **RPA1130** | GATGAGAGATGACGACAGGAATCCGAATGAGCTTGGAACTCAGCCATGATTG |  | - |
| **RPA1131** | CGCCTCTCTAAGCTCGTCGTATGAAGGAAACGCCCCACATACGGCCTCAGAC |  | - |
| **RPA1132** | GGCCGTATGTGGGGCGTTTCCTTCATACGACGAGCTTAGAGAGGCGGATATGG |  | - |
| **RPA1133** | CCGCCCCGTCCCGTCCCTGGTCAGCGGGGAATGGCGCCCGCGATCATAATTG |  | - |
| **RPA1134** | CAATTATGATCGCGGGCGCCATTCCCCGCTGACCAGGGACGGGACGGGGCGGAGC |  | - |
| **RPA1135** | GAGCTTGGATTCTGCGTTTGTTTCCGTCTACGAACTCCCAGCCGGCCCCGGTCCCTATAG |  | - |
| **RPA884** | GCCGGCTGGGAGTTCGTAGACGGAAACAAACGCAGAATCCAAGCTCAGGCGATTCCGGTGGC |  | - |
| **RPA885** | GGCCACCCCCGACGCCAAAGCACAAACCGGTTAAACACTTTGCCATCGATCGAG |  | - |
| **RPA886** | GGCAAAGTGTTTAACCGGTTTGTGCTTTGGCGTCGGGGGTGGCCGCTTATAAGAACGGCTCC |  | - |
| **RPA887** | CATACCATCGTGGACGTGCGTGGATTGCCGGTGAAATTGCTGCTAACGCCGAGC |  | - |
| **RPA888** | GCGTTAGCAGCAATTTCACCGGCAATCCACGCACGTCCACGATGGTATGAATTTTGGTGCTCCGTCC |  | - |
| **RPA889** | GGAAGGGCTCGGAGTTGTGGTAATCTATGTATCCTGGCGCGCGCGCATGACCGCACAGACTATCGGGTTGTCGC |  | - |

**Table S1 C:** Plasmids used in this study.

| Plasmid | Relevant characteristic (s) | References and/or source |
| --- | --- | --- |
| pORFM | Universal in-frame deletion/in-frame fusion vector for GalK based counter selection; *npt galk tetR mobRK2* | (7) |
| pORFM-∆*mamL* _MSR-1_ | pORFM based plasmid used for chromosomal deletion of *mamL* (Locus_tag: MSR1-03390) in MSR-1. | This study |
| pORFM-∆*mamE* _MSR-1_ | pORFM based plasmid used for chromosomal deletion of *mamE* (Locus_tag: MSR1-03360) in MSR-1. | This study |
| pORFM-∆*mamO* _MSR-1_ | pORFM based plasmid used for chromosomal deletion of *mamE* (Locus_tag: MSR1-03420) in MSR-1. | This study |
| pORFM-∆*mamAB*op*_MSR-1_* | pORFM based plasmid used for chromosomal deletion of *mamAB*op (Locus_tag: MSR1-03340-MSR1-03500) in ∆A13 _MSR-1_. | This study |
| pBAM-P*_mamDC_*_45_-*mamC*-*egfp* | Tn5-based integrative plasmid, Kn^R^. Amp^R^, *oriR6K*, *tnpA* | (8) |
| pBAM-Ptet-*popZ*-HL-*egfp* | Tn5-based integrative plasmid harboring an anhydrotetracycline inducible promoter. | (9) |
| pBAM-Tn5-P*_mamDC_*_45_-*egfp* (N-ter) | Tn5-based integrative plasmid harboring a constitutive promoter P*_mamDC_*_45_-*egfp* (for *N*-terminus fusion) | This study |
| pBAM-Tn5-P*_mamDC_*_45_-*egfp* (C-ter) | Tn5-based integrative plasmid harboring a constitutive promoter P*_mamDC_*_45_-x-*egfp* (for *C*-terminus fusion, x stands for gene) | This study |
| pBAM-Tn5-P*_mamH_*- *egfp* (C-ter) | Tn5-P*_mamH_*-based integrative plasmid; Kn^R^, Amp^R^ | This study |
| pBAM-Tn5-P*_mamH_*-*mamL* _MSR-1_ | Tn5-based integrative plasmid harboring a constitutive P*_mamH_*-*mamL* _MSR-1_expression cassette. | This study |
| pBAM-Tn5-P*_mamH_*-*mamQ* _MSR-1_ | Tn5-based integrative plasmid harboring a constitutive P*_mamH_*-*mamQ* _MSR-1_expression cassette. | This study |
| pBAM-Tn5-P*_mamH_*-*mamB* _MSR-1_ | Tn5-based integrative plasmid harboring a constitutive P*_mamH_*-*mamB* _MSR-1_expression cassette. | This study |
| pBAM-Tn5-P*_mamH_*-*mamM* _MSR-1_ | Tn5-based integrative plasmid harboring a constitutive P*_mamH_*-*mamM* _MSR-1_expression cassette. | This study |
| pBAM-Tn5-P*_mamH_*-*mamE* _MSR-1_ | Tn5-based integrative plasmid harboring a constitutive P*_mamH_*-*mamE* _MSR-1_expression cassette. | This study |
| pBAM-Tn5-P*_mamH_*-*mamO* _MSR-1_ | Tn5-based integrative plasmid harboring a constitutive P*_mamH_*-*mamO* _MSR-1_expression cassette. | This study |
| pBAM-Tn5-P*_mamH_*-*mamL*_AMB-1_ | Tn5-based integrative plasmid harboring a constitutive P*_mamH_*-*mamL*_AMB-1_ expression cassette. | This study |
| pBAM-Tn5-P*_mamH_*-*mamQ*_AMB-1_ | Tn5-based integrative plasmid harboring a constitutive P*_mamH_*-*mamQ*_AMB-1_ expression cassette. | This study |
| pBAM-Tn5-P*_mamH_*-*mamB*_AMB-1_ | Tn5-based integrative plasmid harboring a constitutive P*_mamH_*-*mamB*_AMB-1_ expression cassette. | This study |
| pBAM-Tn5-P*_mamH_*-*mamM*_AMB-1_ | Tn5-based integrative plasmid harboring a constitutive P*_mamH_*-*mamM*_AMB-1_ expression cassette. | This study |
| pBAM-Tn5-P*_mamH_*-*mamE*_AMB-1_ | Tn5-based integrative plasmid harboring a constitutive P*_mamH_*-*mamE*_AMB-1_ expression cassette. | This study |
| pBAM-Tn5-P*_mamH_*-*mamO*_AMB-1_ | Tn5-based integrative plasmid harboring a constitutive P*_mamH_*-*mamO*_AMB-1_ expression cassette. | This study |
| pBAM-Tn5-P*_mamH_*-*mamL*_MV-1_ | Tn5-based integrative plasmid harboring a constitutive P*_mamH_*-*mamL*_MV-1_ expression cassette. | This study |
| pBAM-Tn5-P*_mamH_*-*mamQ*_MV-1_ | Tn5-based integrative plasmid harboring a constitutive P*_mamH_*-*mamQ*_MV-1_ expression cassette. | This study |
| pBAM-Tn5-P*_mamH_*-*mamB*_MV-1_ | Tn5-based integrative plasmid harboring a constitutive P*_mamH_*-*mamB*_MV-1_ expression cassette. | This study |
| pBAM-Tn5-P*_mamH_*-*mamM*_MV-1_ | Tn5-based integrative plasmid harboring a constitutive P*_mamH_*-*mamM*_MV-1_ expression cassette. | This study |
| pBAM-Tn5-P*_mamH_*-*mamE*_MV-1_ | Tn5-based integrative plasmid harboring a constitutive P*_mamH_*-*mamE*_MV-1_ expression cassette. | This study |
| pBAM-Tn5-P*_mamH_*-*mamO*_MV-1_ | Tn5-based integrative plasmid harboring a constitutive P*_mamH_*-*mamO*_MV-1_ expression cassette. | This study |
| pBAM-Tn5-P*_mamH_*-*mamL*_MC-1_ | Tn5-based integrative plasmid harboring a constitutive P*_mamH_*-*mamL*_MC-1_ expression cassette. | This study |
| pBAM-Tn5-P*_mamH_*-*mamQ*_MC-1_ | Tn5-based integrative plasmid harboring a constitutive P*_mamH_*-*mamQ*_MC-1_ expression cassette. | This study |
| pBAM-Tn5-P*_mamH_*-*mamB*_MC-1_ | Tn5-based integrative plasmid harboring a constitutive P*_mamH_*-*mamB*_MC-1_ expression cassette. | This study |
| pBAM-Tn5-P*_mamH_*-*mamM*_MC-1_ | Tn5-based integrative plasmid harboring a constitutive P*_mamH_*-*mamM*_MC-1_ expression cassette. | This study |
| pBAM-Tn5-P*_mamH_*-*mamE*_MC-1_ | Tn5-based integrative plasmid harboring a constitutive P*_mamH_*-*mamE*_MC-1_ expression cassette. | This study |
| pBAM-Tn5-P*_mamH_*-*mamO*_MC-1_ | Tn5-based integrative plasmid harboring a constitutive P*_mamH_*-*mamO*_MC-1_ expression cassette. | This study |
| pBAM-Tn5-P*_mamH_*-*mamL*_RS-1_ | Tn5-based integrative plasmid harboring a constitutive P*_mamH_*-*mamL*_RS-1_ expression cassette. | This study |
| pBAM-Tn5-P*_mamH_*-*mamQ*_RS-1_ | Tn5-based integrative plasmid harboring a constitutive P*_mamH_*-*mamQ*_RS-1_ expression cassette. | This study |
| pBAM-Tn5-P*_mamH_*-*mamB*_RS-1_ | Tn5-based integrative plasmid harboring a constitutive P*_mamH_*-*mamB*_RS-1_ expression cassette. | This study |
| pBAM-Tn5-P*_mamH_*-*mamM*_RS-1_ | Tn5-based integrative plasmid harboring a constitutive P*_mamH_*-*mamM*_RS-1_ expression cassette. | This study |
| pBAM-Tn5-P*_mamH_*-*mamE*_RS-1_ | Tn5-based integrative plasmid harboring a constitutive P*_mamH_*-*mamE*_RS-1_ expression cassette. | This study |
| pBAM-Tn5-P*_mamH_*-*mamO*_RS-1_ | Tn5-based integrative plasmid harboring a constitutive P*_mamH_*-*mamO*_RS-1_ expression cassette. | This study |
| pBAM-Tn5-P*_mamDC_*_45_-*egfp*-*mamL*_MV-1_ | Tn5-based integrative plasmid harboring a constitutive P*_mamDC_*_45_-*egfp*-*mamL*_MV-1_ expression cassette. | This study |
| pBAM-Tn5-P*_mamDC_*_45_-*egfp*-*mamQ*_MV-1_ | Tn5-based integrative plasmid harboring a constitutive P*_mamDC_*_45_-*egfp*-*mamQ*_MV-1_ expression cassette. | This study |
| pBAM-Tn5-P*_mamDC_*_45_-*mamB*_MV-1_-*egfp* | Tn5-based integrative plasmid harboring a constitutive P*_mamDC_*_45_-*mamB*_MV-_1-*egfp* expression cassette. | This study |
| pBAM-Tn5-P*_mamDC_*_45_-*mamM*_MV-1_-*egfp* | Tn5-based integrative plasmid harboring a constitutive P*_mamDC_*_45_-*mamM*_MV-_1-*egfp* expression cassette. | This study |
| pBAM-Tn5-P*_mamDC_*_45_-*egfp*-*mamE*_MV-1_ | Tn5-based integrative plasmid harboring a constitutive P*_mamDC_*_45_-*egfp*-*mamE*_MV-1_ expression cassette. | This study |
| pBAM-Tn5-P*_mamDC_*_45_-*egfp*-*mamO*_MV-1_ | Tn5-based integrative plasmid harboring a constitutive P*_mamDC_*_45_-*egfp*-*mamO*_MV-1_ expression cassette. | This study |
| pBAM-Tn5-P*_mamDC_*_45_-*egfp*-*mamL*_MC-1_ | Tn5-based integrative plasmid harboring a constitutive P*_mamDC_*_45_-*egfp*-*mamL*_MC-1_ expression cassette. | This study |
| pBAM-Tn5-P*_mamDC_*_45_-*egfp*-*mamQ*_MC-1_ | Tn5-based integrative plasmid harboring a constitutive P*_mamDC_*_45_-*egfp*-*mamQ*_MC-1_ expression cassette. | This study |
| pBAM-Tn5-P*_mamDC_*_45_-*mamB*_MC-1_-*egfp* | Tn5-based integrative plasmid harboring a constitutive P*_mamDC_*_45_-*mamB*_MC-1_-*egfp* expression cassette. | This study |
| pBAM-Tn5-P*_mamDC_*_45_-*mamM*_MC-1_-*egfp* | Tn5-based integrative plasmid harboring a constitutive P*_mamDC_*_45_-*mamM*_MC-1_-*egfp* expression cassette. | This study |
| pBAM-Tn5-P*_mamDC_*_45_-*egfp*-*mamE*_MC-1_ | Tn5-based integrative plasmid harboring a constitutive P*_mamDC_*_45_-*egfp*-*mamE*_MC-1_ expression cassette. | This study |
| pBAM-Tn5-P*_mamDC_*_45_-*egfp*-*mamO*_MC-1_ | Tn5-based integrative plasmid harboring a constitutive P*_mamDC_*_45_-*egfp*-*mamO*_MC-1_ expression cassette. | This study |
| pBAM-Tn5-P*_mamDC_*_45_-*egfp*-*mamL*_RS-1_ | Tn5-based integrative plasmid harboring a constitutive P*_mamDC_*_45_-*egfp*-*mamL*_RS-1_ expression cassette. | This study |
| pBAM-Tn5-P*_mamDC_*_45_-*egfp*-*mamQ*_RS-1_ | Tn5-based integrative plasmid harboring a constitutive P*_mamDC_*_45_-*egfp*-*mamQ*_RS-1_ expression cassette. | This study |
| pBAM-Tn5-P*_mamDC_*_45_-*mamB*_RS-1-_*egfp* | Tn5-based integrative plasmid harboring a constitutive P*_mamDC_*_45_-*mamB*_RS-1_-*egfp* expression cassette. | This study |
| pBAM-Tn5-P*_mamDC_*_45_-*mamO*_RS-1-_*egfp* | Tn5-based integrative plasmid harboring a constitutive P*_mamDC_*_45_-*mamM*_RS-1_-*egfp* expression cassette. | This study |
| pBAM-Tn5-P*_mamDC_*_45_-*egfp*-*mamO*_RS-1_ | Tn5-based integrative plasmid harboring a constitutive P*_mamDC_*_45_-*egfp*-*mamE*_RS-1_ expression cassette. | This study |
| pBAM-Tn5-P*_mamDC_*_45_-*egfp*-*mamE*_RS-1_ | Tn5-based integrative plasmid harboring a constitutive P*_mamDC_*_45_-*egfp*-*mamO*_RS-1_ expression cassette. | This study |
| pTps-XY | MycoMar transposase gene (*tps*) based integrative plasmid containing *mamXY*op from *MSR-1;* used to construct pTps-Kn^R^-RPA. | (10) |
| pBAMII-Tn7 | Site-specific Tn7-based insertion vector; *tnsABCD*, *p15A*, *oriT*, Kn^R^, Cm^R^; Kn^R^-UNS1-UNSX containing Multiple cloning sites used to construct pTps-Kn^R^-RPA | Dr. Renè Uebe (unpublished plasmid, manuscript in preparation) |
| pTps-Kn^R^-RPA | MycoMar transposase gene *(tps)* based integrative plasmid*; ptps, p15A, oriT,* Kn^R^, UNS-UNSX. | This study |
| pSC101-BAD-gbaA | TcR, replicative plasmid containing redα/redβ recombinases under the control of a L-arabinose inducible promoter for recombineering. | (11) |
| pTPs-Cm^R^-RPA | MycoMar transposase gene *(tps)* based integrative plasmid*; ptps, p15A, oriT,* Cm^R^; Kn^R^ exchanged with Cm^R^ in pTps-Kn^R^-RPA by RedET recombineering. | This study |
| pTps-KnR-P*mamG*-*mamB*_MV-1_-P*mamH*-*mamM*_MV-1_ | *Tps-*based integrative plasmid used for co-expression of *mamB* and *mamM* from MV-1. | This study |
| pTps-KnR-P*mamG*-*mamB*_MC-1_-P*mamH*-*mamM*_MC-1_ | *Tps-*based integrative plasmid used for co-expression of *mamB* and *mamM* from MC-1. | This study |
| pTps-KnR-P*mamG*-*mamB*_RS-1_-P*mamH*-*mamM*_RS-1_ | *Tps-*based integrative plasmid used for co-expression of *mamB* and *mamM* from RS-1. | This study |
| pTps-KnR-*mamAB*_AMB-1_ | *Tps-*based integrative plasmid harboring a constitutive native *mamAB*op from AMB-1. | This study |
| pTps-KnR-*mamAB*_MV-1_ | *Tps-*based integrative plasmid harboring a constitutive native *mamAB*op from MV-1. | This study |
| pTps-KnR-P_MSR-1_-*mamAB*_MV-1_-RG | *Tps-*based integrative plasmid harboring sub-divided (three) *mamAB*op from MV-1, each operon fused transcriptionally to reporter genes encoding mCherry, mTurqoise2, omNeonGreen. | This study |
| pTps-CmR-P*_mamG_*-*mamDFHK*op_MV-1_ | *Tps-*based integrative plasmid harboring an accessory operon (*mamDFHK*) from MV-1. | This study |
| pAG416Gal-ccdB | Centromeric *URA3, attR1-PGAL-ccdB-attR2* | (12) |
| pTps-TAR-RPA | *Tps-*based integrative plasmid used for TAR cloning; *tps, p15A, oriT, Kn^R^, CEN6/URA3* | This study |
| pTps-MAG_AMB-1_ | *Tps-*TAR-RPA based integrative plasmid harboring magnetosome associated gene clusters (five operons) from MAI of AMB-1. | This study |
|  |  |  |

References

1. Schultheiss D, Schüler D. 2003. Development of a genetic system for Magnetospirillum gryphiswaldense. Arch Microbiol 179:89–94. doi:10.1007/s00203-002-0498-z.

2. Lohße A, Borg S, Raschdorf O, Kolinko I, Tompa E, Pósfai M, Faivre D, Baumgartner J, Schüler D. 2014. Genetic dissection of the mamAB and mms6 operons reveals a gene set essential for magnetosome biogenesis in Magnetospirillum gryphiswaldense. J Bacteriol 196:2658–2669. doi:10.1128/JB.01716-14.

3. Uebe R, Keren-Khadmy N, Zeytuni N, Katzmann E, Navon Y, Davidov G, Bitton R, Plitzko JM, Schüler D, Zarivach R. 2018. The dual role of MamB in magnetosome membrane assembly and magnetite biomineralization. Mol Microbiol 107:542–557. doi:10.1111/mmi.13899.

4. Uebe R, Junge K, Henn V, Poxleitner G, Katzmann E, Plitzko JM, Zarivach R, Kasama T, Wanner G, Pósfai M, Böttger L, Matzanke B, Schüler D. 2011. The cation diffusion facilitator proteins MamB and MamM of Magnetospirillum gryphiswaldense have distinct and complex functions, and are involved in magnetite biomineralization and magnetosome membrane assembly. Mol Microbiol 82:818–835. doi:10.1111/j.1365-2958.2011.07863.x.

5. Lohsse A, Ullrich S, Katzmann E, Borg S, Wanner G, Richter M, Voigt B, Schweder T, Schüler D. 2011. Functional analysis of the magnetosome island in Magnetospirillum gryphiswaldense: the mamAB operon is sufficient for magnetite biomineralization. PLoS One 6:e25561. doi:10.1371/journal.pone.0025561.

6. Baker Brachmann C, Davies A, Cost GJ, Caputo E, Li J, Hieter P, Boeke JD. 1998. Designer deletion strains derived from Saccharomyces cerevisiae S288C: A useful set of strains and plasmids for PCR-mediated gene disruption and other applications. Yeast 14:115–132. doi:10.1002/(SICI)1097-0061(19980130)14:2<115:AID-YEA204>3.0.CO;2-2.

7. Raschdorf O, Plitzko JM, Schüler D, Müller FD. 2014. A tailored galK counterselection system for efficient markerless gene deletion and chromosomal tagging in Magnetospirillum gryphiswaldense. Appl Environ Microbiol 80:4323–4330. doi:10.1128/AEM.00588-14.

8. Borg S, Hofmann J, Pollithy A, Lang C, Schüler D. 2014. New vectors for chromosomal integration enable high-level constitutive or inducible magnetosome expression of fusion proteins in Magnetospirillum gryphiswaldense. Appl Environ Microbiol 80:2609–2616. doi:10.1128/AEM.00192-14.

9. Pfeiffer D, Toro-Nahuelpan M, Bramkamp M, Plitzko JM, Schüler D. 2019. The Polar Organizing Protein PopZ Is Fundamental for Proper Cell Division and Segregation of Cellular Content in Magnetospirillum gryphiswaldense. mBio 10. doi:10.1128/mBio.02716-18.

10. Kolinko I, Lohße A, Borg S, Raschdorf O, Jogler C, Tu Q, Pósfai M, Tompa E, Plitzko JM, Brachmann A, Wanner G, Müller R, Zhang Y, Schüler D. 2014. Biosynthesis of magnetic nanostructures in a foreign organism by transfer of bacterial magnetosome gene clusters. Nat Nanotechnol 9:193–197. doi:10.1038/NNANO.2014.13.

11. Wang H, Li Z, Jia R, Hou Y, Yin J, Bian X, Li A, Müller R, Stewart AF, Fu J, Zhang Y. 2016. RecET direct cloning and Redαβ recombineering of biosynthetic gene clusters, large operons or single genes for heterologous expression. Nat Protoc 11:1175–1190. doi:10.1038/nprot.2016.054.

12. Alberti S, Gitler AD, Lindquist S. 2007. A suite of Gateway cloning vectors for high-throughput genetic analysis in Saccharomyces cerevisiae. Yeast 24:913–919. doi:10.1002/yea.1502.
